# Genetic Variability of Ethiopian Chickpea (*Cicer arietinum* L.) Landraces for Acid Soil Tolerance

**DOI:** 10.3390/plants14030311

**Published:** 2025-01-21

**Authors:** Hawi Negusse, Teklehaimanot Haileselassie, Mulatu Geleta, Kassahun Tesfaye

**Affiliations:** 1 Institute of Biotechnology, Addis Ababa University, Addis Ababa 1000, Ethiopia; teklehaimanot.hselassie@aau.edu.et (T.H.); kassahun.tesfaye@aau.edu.et (K.T.); 2 Bio and Emerging Technology Institute (BETin), Addis Ababa 1000, Ethiopia; 3 Department of Plant Breeding, Swedish University of Agricultural Sciences, 23422 Alnarp, Sweden; mulatu.geleta.dida@slu.se

**Keywords:** acidic-soil tolerance, *Cicer arietinum*, lime requirement, soil acidity, yield stability index

## Abstract

Chickpea is among the major legume crops grown globally. In Ethiopia, it plays a vital role in the food security and economic stability of smallholder farmers. However, its production is often hampered by abiotic factors, particularly soil acidity, which is a major yet often overlooked challenge. Using tolerant genotypes alone or combined with soil amendments is a sustainable approach to improving chickpea production in acidic soils. Hence, the present study assessed the genetic variation of 64 Ethiopian chickpea accessions for acidic-soil tolerance using simple lattice design-based field experiments with two replications at two sites with acidic soil, Emdebir and Holetta. The study revealed significant genetic variation among the evaluated accessions for acid soil tolerance. The study also identified tolerant and high-yielding chickpea accessions with a high yield stability index (YSI) at both test sites. The landrace ETC_B_1_2016 exhibited the highest number of primary branches per plant (NPB), number of pods per plant (NPP), and total seed yield (TSY) at the Emdebir acidic soil trial. At the Holetta acidic soil trial, the landrace ETC_41237 recorded the highest TSY, followed by ETC_K_3_2016 and ETC_B_1_2016, while Akaki had the least. In addition, 14 accessions had the highest TSY and YSI at the Emdebir site, while 16 had the highest YSI at the Holetta site. Notably, NPP displayed the strongest positive correlation with TSY at both sites, irrespective of lime application. Higher genetic variance and broad-sense heritability observed for NPP, hundred-seed weight (HSW), and TSY suggest that genetic factors mainly influence these traits and are more likely to improve through selection. The identified acid-tolerant and high-yielding accessions could be considered for direct cultivation in areas with acidic soils, potentially increasing chickpea productivity. Additionally, these accessions can be crossbred with existing improved varieties to enhance their adaptability to acidic soils, ultimately contributing to food security in regions affected by soil acidity.

## 1. Introduction

Chickpea (*Cicer arietinum* L.) is one of the most cultivated legume crops in the world, ranking second after the common bean (*Phaseolus vulgaris*) [1] with a wide distribution in the tropics, subtropics, and temperate regions [2]. It is believed to have originated in present-day southeastern Turkey and the surrounding regions of Syria [3]. Globally, it is produced in more than 50 countries on approximately 15 million hectares of land, with a total production of 15.87 million tonnes and an average productivity of 1.06 tonnes per hectare [1]. India stands out as the largest producer country, accounting for 78% of global chickpea production, followed by Australia (6%), Ethiopia (3%), Turkey (3%), and Myanmar (3%) [1].

Chickpea is the most important grain legume in Ethiopia, crucial for the food security and economic stability of smallholder farmers. It is extensively produced across the highlands and semi-arid regions of the country at altitudes ranging from 1400 to 2300 m a.s.l. and annual average rainfall ranging from 700 to 2000 mm [4]. Currently, it is cultivated on 228,420 hectares of land, resulting in an annual production of 478,212 tonnes, and an average productivity of 2.094 tonnes per hectare, higher than the global average [1]. This makes it the third most widely cultivated legume crop in the country in terms of area coverage and production volume, following faba and haricot beans [5].

Chickpea is a versatile crop with substantial socioeconomic significance for smallholder farmers worldwide. It is high in protein, fiber, mineral, and vitamin contents, making it an essential nutritional staple, especially in areas with limited access to meat [6,7]. The straw is also rich in digestible crude protein, making it suitable for animal feed [7,8]. As a legume crop, it plays a crucial role in crop rotation systems that involve cereals, such as wheat and tef, mitigating pest and disease pressures and enhancing soil fertility through nitrogen fixation, thereby promoting sustainable agricultural practices [8,9]. Furthermore, it generates income for millions of smallholder farmers, particularly in developing nations, thereby reducing poverty and improving rural livelihood [4,8]. Chickpea is one of the key export commodities in Ethiopia, second only to white pea beans, accounting for nearly 25% of total pulse export earnings [9,10].

Despite its extensive nutritional, agronomic, and economic significance, chickpea productivity is profoundly affected by various abiotic factors, including drought, heat, soil salinity, and soil acidity [11]. Among these, soil acidity is the most overlooked abiotic constraint that limits chickpea production and productivity worldwide. More than 50% of the world’s potentially arable lands are acidic, and 60% of these soils exist in tropical and subtropical regions, affecting crop productivity in developing nations [12,13]. In Ethiopia, soil acidity is a significant challenge to major crop productivity [14]. Nearly 43% of potentially arable land is acidic [15,16], 33% of which is associated with aluminum toxicity [14]. Most of these soils exist in regions with high rainfall [17], particularly in the North-Western, South-Western, Southern and Central parts [15].

Acidic soils hinder plant growth by reducing the availability of essential nutrients, such as phosphorous (P), calcium (Ca), magnesium (Mg), and potassium (K) while increasing the solubility of toxic elements such as aluminum (Al), manganese (Mn), and iron (Fe) [18]. Liming is widely used to raise soil pH and amend acidity. However, deep incorporation of lime into sub-soils is challenging and expensive, especially in strongly acidic sub-soils, making it impractical for resource-poor farmers in tropical countries [19]. In contrast, cultivating acid-tolerant crops is a promising strategy for sustainable agriculture, as such crops can thrive under such adverse conditions. This approach reduces reliance on soil amendment inputs and hence promotes ecosystem services.

Genetic variation in acid soil tolerance has been reported in various crops, including common bean [20], soybean [21,22,23], barley [24,25], sorghum [26,27,28], finger millet [29,30,31], durum wheat [32,33], oat [34], and tef [14]. Chickpea, like other grain legumes such as common bean, pigeon pea and pea, is generally regarded as sensitive to soil acidity and Al toxicity [35,36]. However, significant genetic variation in aluminum tolerance exists within the chickpea gene pool [37,38,39,40]. To gain a more comprehensive understanding of chickpea tolerance to acidic soils throughout its growth cycle, evaluating different germplasms under natural soil and climatic conditions across various agro-ecologies is highly desirable. Building on previous germplasm characterization results [37,38], this study aimed at assessing diverse chickpea genetic resources for their tolerance to soil acidity under field conditions using agro-morphological and yield traits as key indicators.

## 2. Results

### 2.1. Genetic Variation Among Chickpea Accessions in Acid Soil Tolerance

Independent analysis of variance (ANOVA) conducted on the data obtained from the Emdebir trial site revealed significant differences among chickpea accessions for all phenotypic traits studied in both lime-treated and lime-untreated soils, except for DTF, which showed no significant differences among accessions in lime-treated soil (Table 1). Similarly, ANOVA for the Holetta trial site showed highly significant differences among the accessions for all traits studied in both lime-treated and lime-untreated soils, indicating sufficient genetic variation among the accessions (Table 2).

Analysis of variance (ANOVA) was conducted on eight relative tolerance indices obtained based on the ratio of absolute values of eight parameters for unlimed versus limed soil. At the Emdebir site, highly significant differences (*p* < 0.001) were observed among chickpea accessions for the relative indices of number of primary branches, number of pods per plant, and seed yield (Table 3).

At the Holetta site, in addition to the traits mentioned above, significant differences were also detected in stand count, days to maturity, and hundred-seed weight (Table 3). These findings indicate substantial genetic variation for acid soil tolerance among the chickpea accessions under study.

At the Emdebir site, the genotypic variance (σ^2^g) for number of pods per plant (NPP), hundred-seed weight (HSW), and total seed yield (TSY) exceeded the environmental variance (σ^2^e) in both lime-treated and untreated experiments (Table 4). In addition, the broad-sense heritability (*H*^2^) for the traits was higher (above 70%) than for other traits studied (Table 4). The phenotypic coefficients of variance (PCV %) and genotypic coefficients of variance (GCV %) at this site ranged from 2.2% for DTM to 36.9% for HSW and from 1.3% for DTF to 33.7% for HSW in the limed experiment, respectively. In the unlimed experiment, the PCV % ranged from 2.3% for DTM to 39.5% for TSY, while the GCV % ranged from 1.6% for DTM to 37.8% for TSY (Table 4).

At the Holetta site, the genotypic variance (σ^2^g) for STC, PH, NPP, HSW, and TSY was higher than the environmental variance (σ^2^e) in the limed experiment. Likewise, in the case of the unlimed experiment, the genotypic variance for the traits STC, DTF, PH, NPP, HSW, and TSY surpassed the environmental variance (σ^2^e) (Table 5). Broad-sense heritability (*H*^2^) of the traits at this site ranged from 36% (for NPB) to 88% (for NPP) and from 37% (for DTM) to 86% (HSW) in the limed and unlimed treatments, respectively.

In the limed trial at the Holetta site, the PCV% and GCV% of the traits ranged from 3.1% for DTM to 54.4% for TSY and 1.9% for DTM to 47.1% for TSY, respectively. In the unlimed trial, the PCV% and GCV% of the traits ranged from 2.5% for DTM to 41.2% for TSY and 1.5% for DTM to 36.8% for HSW, respectively (Table 5).

### 2.2. Effects of Soil Acidity on Agro-Morphological, Yield, and Yield-Related Traits of Chickpea Accessions

**Figure 1:**
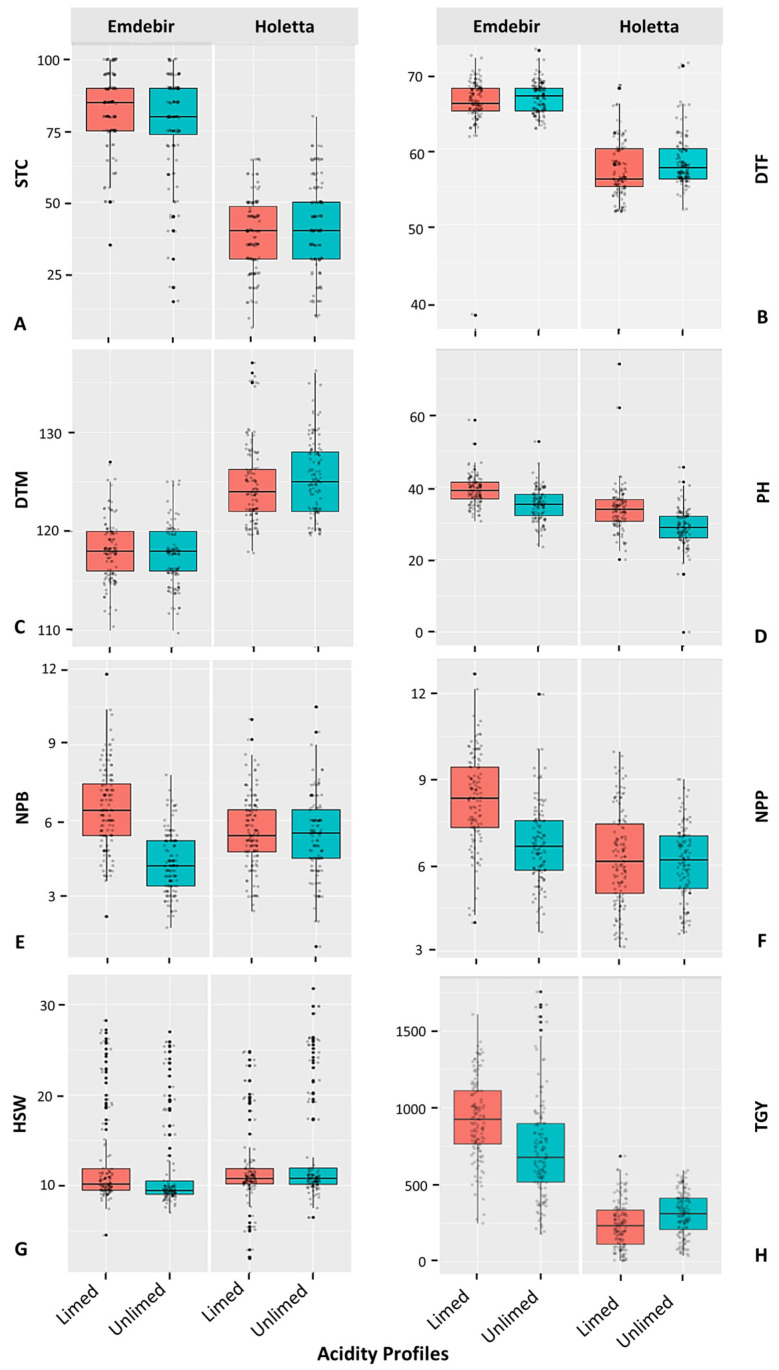
Boxplots of agromorphological and yield traits representing the overall responses of chickpea accessions evaluated at Emdebir and Holetta trial sites under two soil acidity profiles: limed and unlimed conditions. STC = stand count; DTF = days to 50% flowering; DTM = days to maturity; PH = plant height; NPB = number of primary branches per plant; NPP = number of pods per plant; HSW = hundred-seed weight (g); TSY = total seed yield (kg ha^−1^). Horizontal lines in the plot represent the median of each trait’s values.

The comparison of the two trial sites in terms of the average performance of the chickpea accessions revealed that the values of almost all traits were significantly higher at the Emdebir trial site than at the Holetta trial site in both limed and unlimed soils (Table 6; Figure 1; Table S2). At the Emdebir site, all evaluated traits exhibited reduced mean values in the unlimed soil compared to the limed soil, except for days to flowering (DTF) and maturity (DTF), where no significant variation was detected between the two soil treatments.

Similarly, at the Holetta site, lower values were obtained for plant height (PH), number of primary branches per plant (NPB), number of pods per plant (NPP), and total seed yield (TSY) in the unlimed soil than in limed soil. A slight increase in hundred-seed weight (HSW) was also observed in the unlimed soil. However, there was no significant variation in DTF and DTM between the two soil treatments (Table 6; Figure 1; Table S3).

At the Emdebir site, chickpea accessions exhibited a wide range of stand counts (STC), spanning from 43% to 100% and 45% to 100%, with a mean STC of 83% and 78% under limed-treated and lime-untreated experiments, respectively (Table 6). The days to flowering (DTF) also ranged from 52 to 71 and 63 to 73 in the limed and unlimed trials, with an average flowering time of 66 and 67 days, respectively. There is no observed variation between the limed and unlimed experiments for the days to maturity (DTM), with both ranging from 113 to 125 days and a mean DTM of 118 and 117 days, respectively.

Plant height (PH), number of primary branches per plant (NPB), number of pods per plant (NPP) and hundred-seed weight (HSW) of chickpea accessions varied from 32 to 55 cm, 4 to 10, 9 to 59, and 7 to 26 g in the limed trial, while they ranged from 28 to 50 cm, 2 to 7, 6 to 60, and 8 to 26 g in the unlimed trial with a mean of 39 cm, 6, 35, and 13 g and 35 cm, 4, 25 and 12 g, respectively (Table 6).

Interestingly, the highest range of variation was recorded for total seed yield (TSY) under both limed and unlimed trials ranging from 290 to 1519 kg ha^−1^ and 211 to 1657 kg ha^−1^, with an average of 926 and 730 kg ha^−1^, respectively. The highest TSY in the limed and unlimed trials was achieved by the advanced line DZ-2012-CK-0032 and the landrace ETC_B_1_2016. In contrast, the lowest TSY was recorded for the landrace ETC_S_3_2016 (290 kg ha^−1^) and advanced line DZ-2012-CK-0237 (211 kg ha^−1^), respectively (Table S2).

At the Holetta trial site, chickpea accessions showed large variations in PH, NPB, NPP, HSW, and TSY in both limed and unlimed trials. In the limed trial, PH, NPB, NPP, HSW, and TSY ranged from 26 to 68 cm, 3 to 9, 2 to 44, 2 to 25 g, and 13 to 1189 kg ha^−1^, with an average of 34 cm, 6, 22,12 g, 239 kg ha^−1^, respectively. In the unlimed trial, the ranges were from 12 to 42 cm for PH, 2 to 9 for NPB, 5 to 37 for NPP, 7 to 30 g for HSW, and 64 to 534 kg ha^−1^ for TSY, with average values of 29 cm, 5, 21, 13 g, and 309 kg ha^−1^, respectively.

### 2.3. Correlations Between Traits

**Figure 2:**
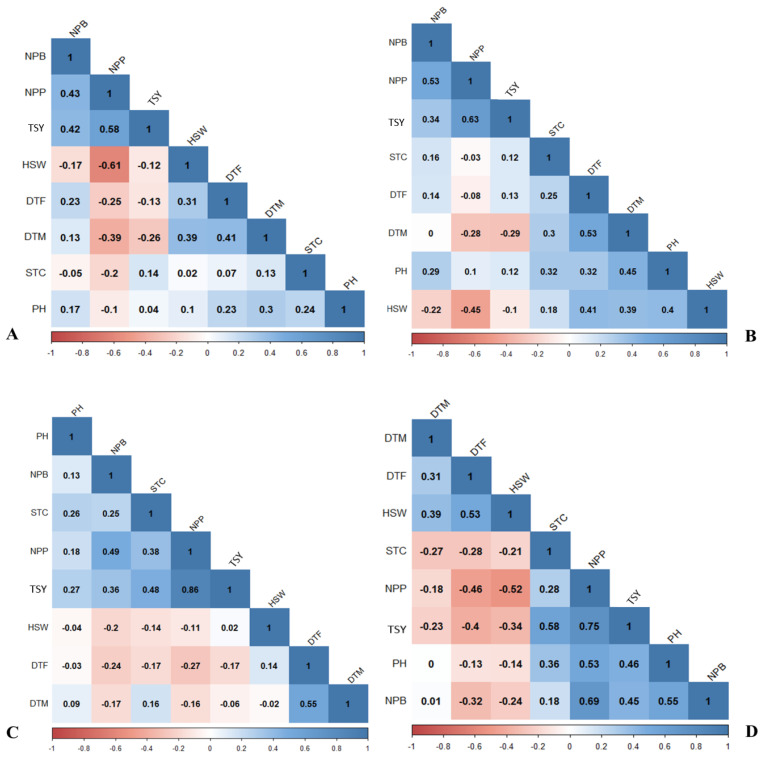
Correlation among eight phenotypic traits of 64 chickpea accessions evaluated under (**A**) lime-treated and (**B**) unlimed conditions at the Emdebir trial site and (**C**) lime-treated and (**D**) unlimed conditions at the Holetta trial site. STC = stand count; DTF = days to 50% flowering; DTM = days to maturity; PH = plant height; NPB = number of primary branches per plant; NPP = number of pods per plant; HSW = hundred-seed weight; and TSY = total seed yield.

Pearson’s correlation analysis was conducted to assess the relationship among the eight agro-morphological and yield-related traits of chickpea accessions across the two experimental sites. The analysis revealed significant positive and negative correlations of different levels between most pairs of traits. At the Emdebir site, the correlations ranged from weak to moderate in both limed (*r* = −0.1 to 0.61) and unlimed experiments (*r* = −0.1 to 0.63) (Figure 2A,B). On the other hand, at the Holetta site, the correlations were notably stronger for some traits, ranging from weak to strong correlations in both the limed (*r* = −0.11 to 0.86) and unlimed (*r* = −0.13 to 0.75) experiments (Figure 2C,D).

At the Emdebir site, the strongest positive correlation was observed between the number of pods per plant (NPP) and total seed yield (TSY) in both limed (*r* = 0.58, *p* < 0.01) and unlimed (*r* = 0.63, *p* < 0.01) experiments. In the limed trial, moderate positive correlations were detected between days to flowering (DTF) and days to maturity (DTM) (*r* = 0.41), number of primary branches per plant (NPB), and TSY (*r* = 0.42), and NPB and NPP (*r* = 0.43). Similarly, in the unlimed trial, moderate positive correlations were found between DTF and DTM (*r* = 0.53), NPB and NPP (*r* = 0.53), DTM and PH (*r* = 0.45), DTF and HSW (*r* = 0.41), and PH and HSW (*r* = 0.40).

On the other hand, moderate negative correlations were found between NPP and HSW in limed (r = −0.61, *p* < 0.001) and unlimed (r = −0.45, *p* < 0.001) treatments, while weak negative correlations were observed between DTM and NPP (r = −0.39 and r = −0.28), and between DTM and TSY (r = −0.26, *p* < 0.01 and r = −0.29, *p* < 0.01) in limed and unlimed experiments, respectively (Figure 2A,B).

At the Holetta site, the strongest positive correlation was observed between NPP and TSY in both limed (*r* = 0.86, *p* < 0.0001) and unlimed (*r* = 0.75, *p* < 0.001) trials (Figure 2C,D). Additionally, there were moderate positive correlations between NPB and NPP (*r* = 0.49 and *r* = 0.69) and STC and TSY (*r* = 0.48 and *r* = 0.58) in limed and unlimed trials, respectively.

In the unlimed trial, moderate positive associations were found among various agro-morphological, yield and yield-related traits: NPB and TSY (r = 0.45), PH and TSY (r = 0.46), DTF and HSW (r = 0.53), PH and NPB (*r* = 0.55), and PH and NPP (*r* = 0.53) (Figure 2D). In the limed trial, DTF exhibited a moderate positive correlation with DTM (*r* = 0.55), while it showed a weak correlation in the unlimed trial (*r* = 0.31). In contrast, significant negative correlations were found between DTF and NPP (*r* = −0.27 and *r* = −0.46), DTF and TSY (*r* = −0.17 and *r* = −0.4), DTF and NPB (*r* = −0.24 and *r* = −0.32), and NPP and HSW (*r* = −0.11, *p* < 0.05 and *r* = −0.52, *p* < 0.001) in the limed and unlimed experiments, respectively (Figure 2C,D).

### 2.4. Mean Trait Performance of Chickpea Accessions at Two Acidic Soil Environments

In the limed trial at the Emdebir site, the improved varieties Kasech and Dubie had the highest stand count (STC), while the advanced line DZ-2012-CK-20113-2-0042 had the least. The landrace ETC_235035 was an early-flowering type (DTF = 52), while Dhera and Natoli were late-flowering types (DTF = 71) (Table S2). The landrace ETC_BM_2_2016 matured early (DTM = 113), while Dhera and ETC_S_2_2016 matured late (DTF = 125). Dhera exhibited the highest plant height (PH = 55 cm), followed by Kasech (PH = 46 cm) and ETC_41237 (PH = 43 cm), while the advanced line DZ-2012-CK-0237 had the least (PH = 32 cm).

The landrace ETC_A_1_2016 exhibited the highest number of pods per plant (NPP = 59), followed by ETC_WL_1_2016 (NPP = 58), while Dhera and Kasech had the least (NPP = 9). The landrace ETC_41237 had the highest number of primary branches per plant (NPB = 10), followed by the landraces ETC_41175 and ETC_41186 (NPB = 9), while the landraces ETC_41118, ETC_41200, and ETC_S_3_2016 had the least (NPB = 4).

The mean hundred-seed weight (HSW) of chickpea accessions revealed that the advanced line DZ-2012-CK-0032 and the improved variety Ejere had the highest HSW, followed by the varieties Natoli and Dalota, while the least was observed in the landrace ETC_SS_2_2016. The mean total seed yield (TSY) of chickpea accessions indicated that the advanced line DZ-2012-CK-0032 revealed the highest TSY in the limed trial (TSY = 1519 kg ha^−1^), followed by the landraces ETC_41237 (TSY = 1382 kg ha^−1^) and ETC_WL_1_2016 (TSY = 1332 kg ha^−1^), while ETC_S_3_2016 had the least (TSY = 290 kg ha^−1^).

At the Emdebir unlimed trial, the landraces ETC_A_2_2016, ETC_B_1_2016, and ETC_S_3_2016 exhibited the highest STC, while the advanced line DZ-2012-CK-20113-2-0042 had the least. The landrace ETC_41086 flowered early in the unlimed trial (DTF = 63), whereas the landraces ETC_S_2_2016 (DTF = 73), and ETC_S_3_2016 (DTF = 71), and the improved variety Akaki (DTF = 71) flowered late. Dhera exhibited the longest days to maturity (DTM = 125), while the landraces ETC_235398 and ETC_TD_4_2016 had the shortest (DTM = 113). Similar to the limed trial, Dhera exhibited the highest PH in the unlimed trial (PH = 50 cm), followed by Yelebe (PH = 41 cm) and Kasech (PH = 40 cm), while ETC_216853 had the least (PH = 28 cm). The NPB of chickpea accessions revealed that ETC_41237 and ETC_B_1_2016 had the highest branch counts (NPB = 7), whereas ETC_H_6_2016 had the least (NPB = 2). The landraces ETC_41175, ETC_41200, ETC_208985, ETC_B_2_2016, and ETC_Il_1_2016 revealed the least HSW (HSW = 8 g), while the improved varieties Ejere demonstrated the highest (HSW = 26 g). The landrace ETC_B_1_2016 revealed the highest NPP (60) and TSY (1657 kg ha^−1^), followed by ETC_HA_2_2016 (NPP = 47 and TSY = 1567 kg ha^−1^), while Kasech and DZ-2012-CK-0237 revealed the least NPP (6) and TSY (211 kg ha^−1^), respectively.

At the Holetta trial site, the landrace ETC_235035 exhibited the highest STC in the unlimed trial, followed by ETC_41224, ETC_GN_1_2016, and ETC_41271. In the limed trial, the landraces ETC_41282 and ETC_HA_2_2016 revealed the highest STC, followed by ETC_215667 and ETC_235398. However, the advanced line DZ-2012-CK-0237 consistently exhibited the least STC in both limed and unlimed trials. The Landraces ETC_41186 and ETC_AM_1_2016 exhibited early DTF in the unlimed trial (DTF = 54), while the advanced line DZ-2012-CK-0237 revealed late (DTF = 71). Similar to the Emdebir site, Dhera exhibited the longest DTM in the Holetta unlimed trial (DTM = 134), while the landrace ETC_41282 had the shortest (DTM = 120). Ejere, Dalota, and ETC_41200 matured early in the limed trial (DTM = 120), while ETC_S_3_2016 matured late (DTM = 136). The landrace ETC_41086 revealed the highest PH in the unlimed soil (PH = 42 cm) followed by ETC_215667 (PH = 37 cm) and ETC_B_1_2016 (PH = 37 cm). In the limed soil, ETC_Il_1_2016 demonstrated the highest PH (PH = 68 cm), followed by the landraces ETC_41086 (PH = 41 cm) and ETC_B_1_2016 (PH = 41 cm). However, the landrace ETC_A_2_2016 consistently exhibited the least PH in the limed and unlimed experiments (PH = 26 cm and PH = 12 cm, respectively). The landrace ETC_215667 revealed the highest NPB and NPP in the Holetta unlimed trial (NPB = 9 and NPP = 37), while ETC_A_2_2016 had the least (NPB = 2 and NPP = 5). The landrace ETC_41237 exhibited the highest TSY in the unlimed trial (TSY = 534 kg ha^−1^) followed closely by ETC_41280 (TSY = 511 kg ha^−1^) and ETC_41265 (TSY = 503 kg ha^−1^), while Akaki had the least (TSY = 64 kg ha^−1^).

In the limed trial, ETC_K_3_2016 had the highest TSY (1189 kg ha^−1^), followed by ETC_41237 (1052 kg ha^−1^) and ETC_41248 (1002 kg ha^−1^) while Kasech had the least (13 kg ha^−1^). It is worth noting that the landrace ETC_B_1_2016 had the longest PH in the unlimed trials of both locations. Additionally, it had the highest NPB and NPP, as well as had the highest TSY at the Emdebir unlimed trial.

### 2.5. Classification of Chickpea Genotypes Using Stress Tolerance Indices

The yield stability index (YSI) of chickpea accessions at the Emdebir site revealed that the landrace ETC_HA_2_2016 exhibited the highest YSI value, followed by ETC_K_3_2016 and ETC_S_3_2016, while ETC_H_6_2016 showed the least. In contrast, at the Holetta site, the improved cultivar Kasech displayed the highest YSI value, followed by the advanced line DZ-2012-CK-0237 and the landrace ETC_41200, while Akaki had the least (Table S4).

Based on the stress tolerance index (STI), the landrace ETC_B_1_2016 showed the highest STI at the Emdebir site, closely followed by ETC_WL_1_2016 and ETC_231330. At the Holetta site, the highest STI was recorded by ETC_41237, followed by ETC_K_3_2016 and ETC_B_1_2016. However, at both locations, the lowest STI was exhibited by the advanced line DZ-2012-CK-0237 (Table S4).

At the Emdebir site, the stress tolerance level (TOL) of Ethiopian chickpea germplasm computed for total seed yield (TSY) under limed and unlimed conditions revealed that the landrace ETC_H_6_2016 exhibited the highest TOL value followed by the advanced line DZ-2012-CK-0032, and the landrace ETC_235031. On the other hand, the landraces ETC_HA_2_2016, ETC_K_3_2016, ETC_41191, ETC_B_1_2016, ETC_Il_1_2016, ETC_S_3_2016, ETC_236462, ETC_41175, ETC_S_2_2016, ETC_B_2_2016, ETC_WL_1_2016, ETC_K_6_2016, ETC_BM_2_2016, and the improved varieties Natoli, and Dhera showed the lowest and negative values of TOL. At the Holetta site, the landrace ETC_235396 exhibited the highest TOL values, followed by ETC_K_3_2016 and ETC_41248, while ETC_41265 exhibited the lowest TOL values, followed by ETC_41280 and ETC_41200. In addition to the mentioned chickpea accessions, Kasech, DZ-2012-CK-0233, ETC_41175, ETC_41086, ETC_41191, DZ-2012-CK-0237, Dhera, ETC_209008, ETC_208985, Dubie, ETC_41186, ETC_BM_2_2016, and ETC_215667 showed negative TOL values at the Holetta trial site.

## 3. Discussion

### 3.1. Variability Among Ethiopian Chickpea Accessions for Acid Soil Tolerance

Soil acidity is one of the most important limitations to agricultural production worldwide. More than half of acidic soils are found in tropical and subtropical regions, affecting crop productivity in areas with the highest population growth and increasing demand for food [13,41]. Despite the toxicity and low fertility of the soil, these regions have favorable topography, adequate temperature, and sufficient moisture for year-round crop production [41]. Hence, by implementing comprehensive management options, such as liming, use of appropriate fertilizers, and using acid-tolerant crops, the productivity of acidic soils can be among the highest in the world [41,42]. Breeding for acid soil tolerance should be thoroughly conducted under a range of conditions, and genotypes that perform well under low nutrient and toxic conditions should be selected to develop materials that incorporate diverse adaptations [19].

The present study assessed the tolerances of 64 Ethiopian chickpea accessions to soil acidity at two acidic sites, Emdebir and Holetta, using agro-morphological and yield traits as key indicators. The study revealed significant differences among chickpea accessions for all traits evaluated in the unlimed acid-stressed trials of both test locations, indicating the presence of considerable genetic variability among the evaluated chickpea accessions for acid soil tolerance. In a comparable study, Alemu and Lule [43] also found significant genetic variability among Desi-type chickpea accessions for acid soil tolerance of Western Ethiopia. Genetic variability for acid soil tolerance has also been previously reported in various crop species, including common bean [20], soybean [21], barley [25,44], durum wheat [32,45] and tef [14].

Notably, chickpea accessions also differed significantly in the limed trials at both test locations, indicating the existence of a significant genetic diversity among the chickpea accessions under study, which provides a broad genetic base for selecting and improving desirable traits. Variability among Ethiopian chickpea landraces for various agro-morphological and yield traits was reported by [46]. Tsehaye et al. [47] also reported variability among various chickpea accessions. Keneni et al. [48] also reported the existence of high genetic diversity in Ethiopian chickpea germplasm accessions using SSR markers.

### 3.2. Traits Association and Heritability of Chickpea Accessions

The association analysis of agronomic and yield traits revealed positive correlations between days to flowering (DTF) and days to maturity (DTM), number of primary branches per plant (NPB) and number of pods per plant (NPP), number of primary branches per plant (NPB) and total seed yield (TSY) and number of pods per plant (NPP) and total seed yield (TSY) at both experimental sites under limed and unlimed conditions. Notably, the association between the number of pods per plant (NPP) and total seed yield (TSY) was the strongest at both sites, regardless of whether lime was applied or not, suggesting that number of pods per plant can be used as selection criteria for improving yield in chickpea. Legesse et al. [20] also stated that seed yield was closely associated with the number of pods per plant in both unlimed and limed soils. Reddy et al. [49] stated that the number of primary branches per plant, pods per plant, and seeds per plant all play a crucial role in increasing the seed yield in chickpea. Hence, the selection and improvement of germplasm based on these characteristics should be prioritized to improve the production potential of chickpea. Ali et al. [50] also reported similar results in chickpea, in which the number of pods per plant revealed a significant and positive correlation with seed yield. Bedassa et al. [21] also found a strong positive correlation between the number of pods per plant and seed yield in the soybean accessions grown on lime-treated and untreated acid soil. Mohammed and Fikre [51] also mentioned that identification and utilization of traits that positively contribute to yield are crucial as they significantly improve the breeding efficiency of chickpea.

On the other hand, negative correlations were observed between DTM and TSY at both the Emdebir and Holetta sites, regardless of lime treatment. Additionally, there were negative correlations between DTF and TSY in both limed and unlimed trials at Holetta, as well as in the limed trial at Emdebir. Furthermore, a negative correlation between HSW and TSY was noted in the limed trial at Emdebir and the unlimed trial at the Holetta site. Similar findings were reported by Gemeda and Gurmu [52] in chickpea, who found negative correlations between days to flowering, days to maturity, and 100-seed weight with seed yield. Mohammed and Fikre [51] also found a negative relationship between seed yield and days to maturity in chickpea. Legesse et al. [20] also reported a negative association between DTM and TSY for common bean in both acid stressed and limed soils.

The genetic variance for traits such as NPP, HSW, and TSY is more than twice the environmental variance at the Emdebir site and more than three times of the environmental variance at the Holetta site, in both with and without lime trials. Additionally, NPP, HSW, and TSY exhibited the highest heritability compared to the remaining traits at both locations and acidity profiles. The combination of the highest heritability and genetic variance suggests that the observed variation in these traits is primarily influenced by genetic factors. As a result, these traits could be improved through selection as they are more stable and consistent than others. Therefore, selecting chickpea accessions based on these traits, whether in acid-stressed or potential soil, may lead to more reliable and improved performance in those locations. Tsehaye et al. [47] reported high heritability along with relatively high values of GCV % and genetic advance as a percentage of mean in chickpea for the traits grain yield, number of pods per plant, and hundred-seed weight. Biru et al. [53] also reported similar results, stating that high heritability combined with high genetic advance was observed for traits HSW, NPP, and TSY in chickpea accessions evaluated under the acidic soils of western Ethiopia, suggesting that these traits can serve as effective tools for phenotypic selection, as they are predominantly governed by additive genes and are less affected by environmental factors. Banik et al. [54] also reported that heritability estimates were high for the number of pods per plant, hundred-seed weight, and seed yield, suggesting that selection for genetic improvement of these traits would be effective in increasing seed yield in chickpea.

### 3.3. Performance of Chickpea Accessions at the Two Acidic Soil Environments

The chickpea accessions responded markedly to the soil acidity at the Emdebir site, which was evident in the overall reduction in plant height (PH), branch count (NPB), number of pods per plant (NPP), hundred-seed weight (HSW), and total seed yield (TSY), which decreased by 9.95%, 31.80%, 28.43%, 6.13%, and 21.13%, respectively, in the unlimed acid-stressed trial compared to the limed trial. Similarly, results from the Holetta site also exhibited an overall decline in the average performance of chickpea accessions for PH, NPB, NPP, and TSY in the unlimed soil compared to limed soil, with the most significant decline observed in TSY (35.35%) and PH (14.87%). This indicates that soil acidity was clearly prevalent at these locations, and applying lime reduces its toxic effects, leading to improved growth and performance of the accessions in the lime-treated soil. In line with our result, Bedassa et al. [21] also reported a significant reduction of 11.91% in PH, 16.06% in NPP, and 13.67% in TSY in soybean accessions evaluated in lime-untreated soil compared to treated soil. Legesse et al. [20] also found an average reduction of 16.7%, 20.2%, 19.1%, and 2.3% in PH, NPP, HSW, and TSY, respectively, of common bean accessions under unlimed soil than limed soil. Similarly, Wayima et al. [33] also reported 18% decline in the grain yield and 28% decline in the biomass of Ethiopian durum wheat landraces at the acidic site compared to the limed site.

In contrast to the toxic effects of acidity, some accessions exhibited significantly better performance in the unlimed trials at both locations than in the limed trial. The landrace ETC_B_1_2016 performed best at the Emdebir acid-stressed trial, producing 1657 kg ha^−1^ of TSY, significantly higher than the TSY obtained in the limed trial, which was 1171 kg ha^−1^. Besides, ETC_B_1_2016 also exhibited the highest NPP count in the acid-stressed trial, surpassing the maximum NPP obtained in the limed trial. In addition, 14 chickpea genotypes: ETC_HA_2_2016, ETC_K_3_2016, ETC_41191, ETC_Il_1_2016, ETC_S_3_2016, ETC_236462, ETC_41175, ETC_S_2_2016, Natoli, ETC_B_2_2016, ETC_WL_1_2016, ETC_K_6_2016, ETC_BM_2_2016 and Dhera demonstrated higher TSY in the unlimed acid-stressed trial at Emdebir site compared to the limed trial. Similarly, these accessions also exhibited the highest yield stability index (YSI) or relative total seed yield (RTSY) at the Emdebir site.

Similarly, at the Holetta site, 16 chickpea accessions, ETC_41265, ETC_41280, ETC_41200, Kasech, DZ-2012-CK-0233, ETC_41175, ETC_41086, ETC_41191, DZ-2012-CK-0237, Dhera, ETC_209008, ETC_208985, Dubie, ETC_41186, ETC_BM_2_2016, and ETC_215667, performed remarkably well in the unlimed trial than the limed trial, exhibiting the highest YSI (RTSY) at the Holetta site. In our study, Natoli and the local landrace ETC_WL_2016 performed best at the Emdebir site, while Dubie performed well at the Holetta site.

Notably, all of the best-performing and high-yielding accessions mentioned above, which were found at the Emdebir and Holetta sites, were also identified as tolerant and highly tolerant genotypes in the previous Al tolerance screening experiment conducted in the Ethiopian chickpea germplasm, except the landraces ETC_Il_1_2016 and ETC_K_6_2016, and the improved varieties Dhera and Kasech, which were found to be susceptible in the screening experiment [37,38]. Therefore, given the highest performance exhibited by these chickpea accessions in both Al-stressed nutrient solution and under field conditions, they appear to have natural tolerance or adaptability to acidic soil conditions. This inherent tolerance is, therefore, vital for enhancing chickpea productivity, ultimately contributing to food security in the region. Bedassa et al. [21] also reported an increase in grain yield in some tolerant soybean accessions in unlimed soil compared to limed soil. Our result also aligns with Alemu and Lule [43], who identified the improved cultivar Natoli and the advanced line DZ-2012-CK-0237 as best performers in the acidic soils of western Ethiopia. Tilahun et al. [55] stated that different chickpea accessions respond differently to varying environmental conditions. 

## 4. Materials and Methods

### 4.1. Plant Materials

**Figure 3:**
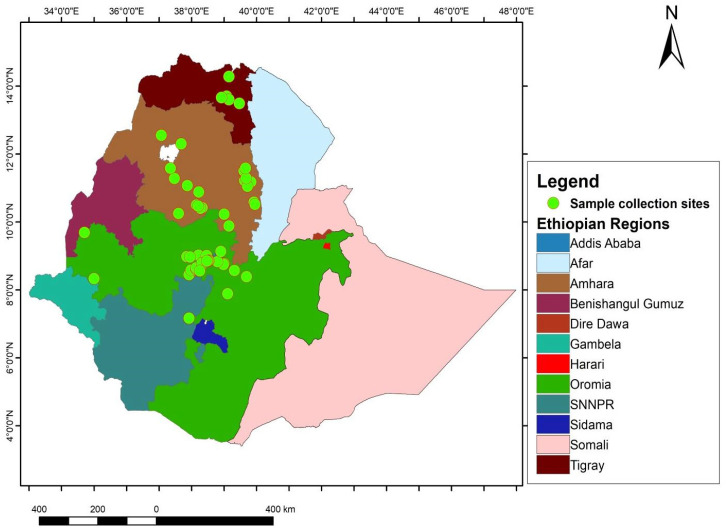
Map of Ethiopia displaying the collection sites of chickpea accessions in different regional states (indicated by green circles) of landrace accessions sourced from EBI and directly collected from farmers’ fields.

A total of 64 chickpea accessions were used in this study. The accessions include 34 gene bank accessions obtained from the Ethiopian Biodiversity Institute (EBI), 18 landraces directly collected from farmers’ fields in various parts of Ethiopia, eight nationally released improved varieties and four advanced lines obtained from the Debrezeit Agricultural Research Center (DZARC), Ethiopia. The accessions were selected based on their performance in hydroponic screening experiments [37,38] and preliminary field trials. Table S1 provides detailed descriptions of the accessions used in this study, and Figure 3 illustrates the sample collection sites.

### 4.2. Soil Sampling and Analysis

Representative soil samples were collected from the topsoil (up to 20 cm deep) using the traverse (diagonal) method with an auger. First, the corners of the field were identified, and then approximately fifteen sub-samples were taken diagonally from each test site. These sub-samples were then thoroughly mixed to create composite samples that capture the overall characteristics of each site. The resulting two composite samples from the two test sites were then sent for soil chemical analysis to JIJE Analytical Testing Service Laboratory, Addis Ababa, Ethiopia, where the soil pH (pHwater and pHKCl), cation exchange capacity (CEC), and exchangeable acidity measurements were quantified. The chemical properties of soil samples collected from two test sites are presented in Table 7. According to the pH scale, both locations have soils classified as strongly acidic [56].

Based on the soil analysis results, the amount of lime (CaCO_3_) required for each experimental site was determined using the formula provided in the lime guidelines for field crops [57]:

(1) $$LR=\frac{EA*0.5*(BS\;desired-BS\;original)}{\left(1-BS\;original\right)}\times \frac{tillage\;depth}{\left(6\right)}$$

where LR = lime requirement, EA = exchangeable acidity, BS original = base saturation at the current pH, and BS desired = base saturation at the desired pH.

The amount of lime determined for each site was applied to the experimental plots 30 days before planting to ensure sufficient incubation time.

### 4.3. Description of the Study Sites

The present study was conducted at two sites in the central highlands of Ethiopia: Emdebir and Holetta (Erob Geba) during the 2018–2019 crop-growing seasons. The Emdebir trial site is located south-west of Addis Ababa, in the Cheha woreda, Guraghe zone of Central Ethiopia Regional State, whereas the Holetta (Erob Geba) trial site is located in the Oromia Regional State, West Shewa Zone, and Welemera woreda.

### 4.4. Experimental Design, Lime Application and Planting

The field trial layout at both sites was an 8 × 8 simple lattice and the experiment comprised two acidity profiles: with and without lime application. The lime application rates were 3946 kg ha^−1^ at Emdebir and 650 kg ha^−1^ at Holetta, as determined using the formula described above. Lime was broadcast uniformly and mixed with the topsoil layer a month before planting. Seeds of each accession were planted in two rows in a 0.6 m^2^ (1 m × 0.6 m) plot, with 30 cm between-row and 10 cm between-plant spacing, in two replications. Planting at Holetta was conducted on 22 September 2018 and at Emdebir on 29 September 2018. Standard agronomic practices were applied at both sites.

### 4.5. Traits Recorded

Eight agro-morphological and yield-related traits were recorded according to the International Board for Plant Genetic Resources (IBPGR) standard descriptors for chickpea [58]. The traits include stand count (STC), days to flowering (DTF), days to maturity (DTM), plant height (PH), number of primary branches per plant (NPB), number of pods per plant (NPP), hundred-seed weight (HSW) and total seed yield (TSY). Days to flowering was recorded as the number of days it took from sowing to the stage when 50% of the plants on a plot had flowered and days to maturity were recorded as the number of days from sowing to the stage at which 90% of the plants on a plot had matured and turned yellow. Plant height was measured in centimeters using a meter rod (2m wooden folding ruler, Zhejiang, China) from the base of the plant to the tip of the uppermost leaf. The number of primary branches and the number of pods per plant were counted manually at maturity. Plant height, the number of primary branches per plant, and the number of pods per plant were calculated from the average of five randomly selected plants.

All accessions were harvested at maturity and air-dried at room temperature. Seed yield was calculated as the weight, in grams, of chickpea seeds harvested from the entire plot followed by converting the values to kilograms per hectare (kg ha^−1^). Hundred-seed weight was scored as the weight in grams of hundred randomly selected chickpea seeds.

Acid soil stress indices were calculated according to the following formula (Fekadu et al. [44]; Narasimhamoorthy et al. [59]):

(2) $$Relative\;Tolerance=\frac{Trait\;response\;at\;unlimed\;soil}{Trait\;response\;at\;limed\;soil}\times 100$$

(3) $$Stress\;Tolerance\;Index(STI)=\frac{\left(SYLDLTP)\;(SYLDLUTP\right)}{(µSYLDLTP)2}$$

(4) $$Stress\;Tolerance\;Level\left(TOL\right)=SYLDLTP-SYLDLUTP$$

(5) $$Yield\;Stability\;Index(YSI)=\frac{\left(TSYLUTP\right)}{TSYLTP}$$

where TSYLTP = total seed yield from the lime treated plot, and TSYLUTP = total seed yield from the lime untreated plot.

### 4.6. Statistical Analysis

Descriptive statistics, such as mean, range, and the standard error of mean, were calculated using the ‘*pastecs*’ package in R version 4.1.1 [60]. Analysis of variance (ANOVA) and estimates of variance components were computed using SAS software version 9.4 (SAS Institute Inc. Cary, NC, USA). After conducting the independent ANOVA for each location, homogeneity of variance was tested using the F-max (Hartley’s) test, computed from the independent ANOVA as the ratio of the larger mean square of error (MSE) for each phenotypic trait to the smaller mean square of error as per the formula below [61].

(6) $$F-max=\frac{Larger\;MSE}{Smaller\;MSE}\leq 3.0$$

Genotypic, environmental and phenotypic variance components and their coefficients of variation were estimated for each location using the methods outlined by [62].

(7) $$Genetic\;variance\;(\sigma^{2}g)=\frac{GMS-MSE}{r}$$

(8) $$Environmental\;variance\;(\sigma^{2}e)=Mean\;square\;of\;error$$

(9) $$Phenotypic\;variance\;(\sigma^{2}p)=\sigma^{2}g\;+\;\sigma^{2}e$$

where GMS = genotype mean square; MSE = mean square of error, and r = replication.

Estimates of phenotypic and genotypic coefficients of variance were computed as follows:

(10) $$Phenotypic\;coefficient\;of\;variance\;(\mathrm{PCV})=\frac{\sigma p\times 100}{µ}$$

(11) $$Genotypic\;coefficient\;of\;variance\;(\mathrm{GCV})=\frac{\sigma g\times 100}{µ}$$

where σp = phenotypic standard deviation, σg = genotypic standard deviation, and µ = grand population mean value of a trait.

Broad-sense heritability (*H*^2^) was calculated according to the formula given below [63,64].

(12) $$H^{2}=\frac{\sigma^{2}g}{\sigma^{2}g+\sigma^{2}e}\times 100$$

## 5. Conclusions

Genetic variation in crop genetic resources used for breeding is often crucial to the success of a breeding program. The present study assessed the genetic variability of 64 chickpea accessions for their potential tolerance to soil acidity. The study revealed the presence of substantial genetic variation among the evaluated chickpea accessions in their tolerance to acidic soil, as shown by the variation in total seed yield and related traits. This provides ample opportunities for genetic improvement of chickpea for acid soil tolerance, either through direct selection or hybridization of genotypes with desirable traits. The study identified chickpea accessions (ETC_B_1_2016, ETC_HA_2_2016, ETC_K_3_2016, ETC_41191, ETC_41175, ETC_41237, and ETC_BM_2_2016) that demonstrated both tolerance and high-yield as well as high yield stability index (YSI) at both test acidic soils sites. Additionally, the landrace ETC_WL_2016 and the improved varieties Natoli and Dhera performed well at the Emdebir unlimed trial site, while the advanced lines DZ-2012-CK-0233 and DZ-2012-CK-0237, as well as the improved varieties Dubie and Dhera, showed the best performance at the Holetta unlimed trial site. Furthermore, the results obtained in this study align with the results obtained through nutrient solution-based screening for Al tolerance. The direct use of these tolerant accessions by farmers in areas with acidic soils may enhance chickpea productivity, thereby improving food security in the area. Additionally, these accessions can serve as valuable resources in breeding programs focused on developing chickpea cultivars with enhanced tolerance and adaptation to acidic soils. The considerable variation observed among the chickpea accessions highlights the potential for obtaining more tolerant accessions through field testing of other chickpea accessions conserved ex situ. When breeding chickpea for tolerance to acidic soils, considering traits with high heritability and genetic variance, such as number of pods per plant and total seed yield, will enable the efficient development of superior cultivars with high productivity in acidic soils.

## Figures and Tables

**Table 1:** Mean square of ANOVA for eight agro-morphological traits of 64 chickpea accessions grown at the Emdebir trial site under lime-treated and lime-untreated soil conditions.

Mean Square									
Traits		STC		DTF		DTM		PH	
Source of variation	df	Limed	Unlimed	Limed	Unlimed	Limed	Unlimed	Limed	Unlimed
Accession	63	227.01 **	291.93 *	11.51 ^NS^	4.76 ***	9.54 ***	10.87 ***	20.79 **	18.96 ***
Replication	1	94.53	5382.03 ***	0.07	10.13 *	56.45 ***	64.70 ***	10.13	543.68 ***
Block (Replication)	14	121.49	389.08 *	10.56	5.84 **	4.94	3.35	12.29	9.89
Error		105.20	152.98	9.95	2.04	3.84	4.12	9.39	7.82
Mean		82.58	78.20	66.34	66.94	117.93	117.38	39.19	35.30
R^2^		0.76	0.81	0.65	0.83	0.83	0.82	0.78	0.85
CV (%)		12.42	15.82	4.76	2.14	1.66	1.73	7.79	7.92
RMSE		10.26	12.37	3.15	1.43	1.96	2.03	3.05	2.80
Traits		NPB		NPP		HSW		TSY	
Source of variation	df	Limed	Unlimed	Limed	Unlimed	Limed	Unlimed	Limed	Unlimed
Accession	63	2.61 ***	1.57 ***	167.85 ***	132.96 ***	41.97 ***	40.88 ***	109955.17 ***	159424.55 ***
Replication	1	36.77 ***	32.50 ***	87.62 *	377.44 ***	9.39	0.16	64575.20	643261.53 ***
Block (Replication)	14	2.77 **	0.82 *	73.49 ***	9.53	2.38	3.67	33560.45 *	8032.96
Error		1.01	0.40	19.91	6.53	3.67	2.98	17970.65	6787.21
Mean		6.45	4.35	35.22	25.12	12.54	11.74	925.88	730.14
R^2^		0.85	0.89	0.93	0.97	0.95	0.96	0.90	0.98
CV (%)		15.60	14.61	12.67	10.18	15.30	14.72	14.48	11.28
RMSE		1.01	0.64	4.46	2.56	1.92	1.73	134.05	82.38

STC = stand count; DTF = days to flowering; DTM = days to maturity; PH = plant height; NPB = number of primary branches per plant; NPP = number of pods per plant; HSW = hundred-seed weight; and TSY = total seed yield. Significance codes = ‘***’ 0.001; ‘**’ 0.01; ‘*’ 0.05; NS, ‘not-significant’; df = degrees of freedom; R^2^ = coefficient of determination; CV = coefficient of variation; RMSE = root mean squared error.

**Table 2:** Mean square of ANOVA for eight agro-morphological traits of 64 chickpea accessions grown at the Holetta trial site under lime-treated and lime-untreated soil conditions.

Mean Square									
Traits		STC		DTF		DTM		PH	
Source of variations	df	Limed	Unlimed	Limed	Unlimed	Limed	Unlimed	Limed	Unlimed
Accession	63	250.62 ***	367.29 ***	17.10 ***	15.68 ***	20.14 **	13.98 **	58.42 ***	40.76 ***
Replication	1	15.13	12.50	10.13	9.57 *	2.53	367.88 ***	57.11 *	12.25
Block (Replication)	14	39.46	94.45 *	10.24	4.77 *	16.33	12.10 *	20.82	23.34 *
Error		41.91	47.98	6.85	2.49	9.26	6.50	12.64	13.25
Mean		39	42	57	58	125	126	34	29
R^2^		0.90	0.93	0.82	0.92	0.78	0.83	0.87	0.82
CV (%)		16.81	16.64	4.56	2.70	2.44	2.03	10.49	12.63
RMSE		6.47	6.93	2.62	1.58	3.04	2.55	3.56	3.64
Traits		NPB		NPP		HSW		TSY	
Source of variations	df	Limed	Unlimed	Limed	Unlimed	Limed	Unlimed	Limed	Unlimed
Accession	63	2.53 **	3.35 ***	161.80 ***	109.42 ***	33.25 ***	49.65 ***	29608.84 ***	28536.48 ***
Replication	1	0.01	3.16	25.56	11.64	13.91	13.78	28025.28 *	830.28
Block (Replication)	14	1.36	1.25	20.50 *	22.24 *	4.60	2.51	8346.88 *	7825.83 *
Error		1.19	0.88	10.37	9.72	4.87	3.79	4221.15	3891.96
Mean		6	5	22	21	12	13	239	309
R^2^		0.78	0.86	0.97	0.95	0.90	0.96	0.93	0.92
CV (%)		19.69	17.40	14.85	15.08	18.97	14.57	27.18	20.20
RMSE		1.09	0.94	3.22	3.12	2.21	1.95	64.97	62.39

STC = stand count; DTF = days to flowering; DTM = days to maturity; PH = plant height; NPB = number of primary branches per plant; NPP = number of pods per plant; HSW = hundred-seed weight; and TSY = total seed yield. Significance codes = ‘***’ 0.001; ‘**’ 0.01; ‘*’ 0.05; df = degrees of freedom; R^2^ = coefficient of determination; CV = coefficient of variation; RMSE = root mean squared error.

**Table 3:** The mean square of ANOVA and R^2^ values for the tolerance indices of chickpea accessions, computed as a ratio of trait value under unlimed versus limed conditions.

Mean Square									
S.No	Trait	Emdebir				Holetta			
		Accession	Mean	SE.M	R^2^	Genotype	Mean	SE.M	R^2^
1	RSTC	0.052 ^NS^	0.97	0.02	0.69	0.58 ***	1.22	0.06	0.87
2	RDTF	0.005 ^NS^	1.01	0.01	0.66	0.003 ^NS^	1.02	0.01	0.68
3	RDTM	0.001 ^NS^	1.00	0.002	0.63	0.002 *	1.01	0.003	0.74
4	RPH	0.010 ^NS^	0.91	0.01	0.69	0.033	0.86	0.02	0.69
5	RNPB	0.066 ***	0.71	0.02	0.82	0.227 ***	1.03	0.04	0.78
6	RNPP	0.168 ***	0.76	0.03	0.84	1.088 ***	1.24	0.09	0.86
7	RHSW	0.023 ^NS^	0.95	0.01	0.64	8.565 ***	1.58	0.20	0.94
8	RTSY	0.272 ***	0.83	0.04	0.89	29.347 ***	2.55	0.41	0.89

RSTC = relative stand count; RDTF = relative days to 50% flowering; RDTM = relative days to maturity; RPH = relative plant height; RNPB = relative number of primary branches per plant; RNPP = relative number of pods per plant; RHSW = relative hundred-seed weight and RTSY = relative total seed yield. Significance codes = ‘***’ 0.001; ‘*’ 0.05; NS, ‘not-significant’; SE.M = standard error of mean; R^2^ = coefficient of determination.

**Table 4:** Estimate of variance components and broad-sense heritability for eight phenotypic traits of 64 chickpea accessions grown at Emdebir site under lime-treated and lime-untreated soil conditions.

	Limed							Unlimed						
Trait	Mean	σ^2^g	σ^2^e	σ^2^p	GCV	PCV	*H* ^2^	Mean	σ^2^g	σ^2^e	σ^2^p	GCV	PCV	*H* ^2^
STC	83	61	105	166	9.41	15.52	0.37	78	69.48	153	222	10.69	19.12	0.31
DTF	66	0.78	10	11	1.34	5.03	0.07	67	1.36	2	3	1.74	2.75	0.40
DTM	118	2.85	4	7	1.43	2.24	0.43	117	3.37	4	7	1.57	2.34	0.45
PH	39	5.74	9	15	6.14	9.93	0.38	35	5.57	8	13	6.74	10.46	0.42
NPB	6	0.80	1	2	14.91	23.57	0.44	4	0.58	0	1	19.09	24.84	0.59
NPP	35	73.97	20	94	24.57	27.70	0.79	25	63.21	7	70	31.80	33.41	0.91
HSW	13	19.16	4	23	33.67	36.89	0.84	12	18.95	3	22	36.27	39.03	0.86
TSY	926	45,992.26	17,971	63,963	23.16	27.31	0.72	730	76,318.67	6787	83,106	37.84	39.49	0.92

σ^2^g = genotypic variance; σ^2^e = environment variance; σ^2^p = phenotypic variance; GCV = genotypic coefficient of variation; PCV = phenotypic coefficient of variation; *H*^2^ = broad-sense heritability; STC = stand count; DTF = days to 50% flowering; DTM = days to maturity; PH = plant height (cm); NPB = number of primary branches per plant, NPP = number of pods per plant; HSW = hundred-seed weight (g); TSY = total seed yield (kg ha^−1^).

**Table 5:** Estimate of variance components and broad-sense heritability for eight phenotypic traits of 64 chickpea accessions grown at Holetta site under lime-treated and lime-untreated soil conditions.

	Limed							Unlimed						
Trait	Mean	σ^2^g	σ^2^e	σ^2^p	GCV	PCV	*H* ^2^	Mean	σ^2^g	σ^2^e	σ^2^p	GCV	PCV	*H* ^2^
STC	39	104.36	41.91	146.27	26.19	31.01	0.71	42	159.65	47.98	207.64	30.08	34.31	0.77
DTF	57	5.13	6.85	11.98	3.97	6.07	0.43	58	6.59	2.49	9.09	4.43	5.20	0.73
DTM	125	5.44	9.26	14.70	1.87	3.07	0.37	126	3.74	6.50	10.24	1.54	2.54	0.37
PH	34	22.89	12.64	35.53	14.07	17.53	0.64	29	13.75	13.25	27.00	12.79	17.92	0.51
NPB	6	0.67	1.19	1.86	13.63	22.74	0.36	5	1.24	0.88	2.11	22.24	29.07	0.59
NPP	22	75.72	10.37	86.09	39.55	42.17	0.88	21	49.85	9.72	59.57	33.62	36.75	0.84
HSW	12	14.19	4.87	19.06	31.39	36.38	0.74	13	22.93	3.79	26.72	36.83	39.76	0.86
TSY	239	12,693.84	4221.15	16,915.00	47.14	54.42	0.75	309	12,322.26	3891.96	16,214.22	35.92	41.21	0.76

σ^2^g = genotypic variance; σ^2^e = environment variance; σ^2^p = phenotypic variance; GCV = genotypic coefficient of variation; PCV = phenotypic coefficient of variation; *H*^2^ = broad-sense heritability; STC = stand count; DTF = days to 50% flowering; DTM = days to maturity; PH = plant height (cm); NPB = number of primary branches per plant, NPP = number of pods per plant; HSW = hundred-seed weight (g); TSY = total seed yield (kg ha^−1^).

**Table 6:** The performance of 64 chickpea accessions evaluated at two sites under lime-treated and lime-untreated soil conditions for eight agro-morphological traits.

	Limed						Unlimed					
	Emdebir			Holetta			Emdebir			Holetta		
Trait	G.M	Range	SE.M	G.M	Range	SE.M	G.M	Range	SE.M	G.M	Range	SE.M
STC	83	43.00–100.00	1.15	39	8.00–65.00	1.14	78	45.00–100.00	1.56	42	10.00–73.00	1.39
DTF	66	52.00–71.00	0.29	57	52.00–67.00	0.34	67	63.00–73.00	0.19	58	54.00–71.00	0.30
DTM	118	113.0–125.00	0.26	125	120.0–136.00	0.36	117	113.00–125.00	0.26	126	120.00–134.00	0.34
PH	39	32.00–55.00	0.36	34	26.0–68.00	0.55	35	28.00–50.00	0.39	29	12.00–42.00	0.47
NPB	6	4.00–10.00	0.14	6	3.00–9.00	0.13	4	2.00–7.00	0.11	5	2.00–9.00	0.14
NPP	35	9.00–59.00	0.95	22	2.00–44.00	0.97	25	6.00–60.00	0.84	21	5.00–37.00	0.75
HSW	13	7.00–26.00	0.47	12	2.00–25.00	0.39	12	8.00–26.00	0.45	13	7.00–30.00	0.52
TSY	926	290.00–1519.00	23.46	239	13.00–1189.00	13.29	730	211.00–1657.00	29.52	309	64.00–534.00	12.37

STC = stand count; DTF = days to 50% flowering; DTM = days to maturity; PH = plant height; NPB = number of primary branches per plant; NPP = number of pods per plant; HSW = hundred-seed weight (g); TSY = total seed yield (kg ha^−1^); G.M. = grand mean; SE.M = standard error of mean.

**Table 7:** The geographical positions and physico-chemical properties of the soils of the two test sites.

Parameters	Emdebir	Holetta
Latitude	08°09′01.420′ N	09°7′37.0668″ N
Longitude	037°55′00.938″ E	38°26′36.4128″ E
Altitude (m.a.s.l.)	2024	2595
% moisture	5.82	5.26
pH_water_	4.65	4.85
pH_KCl_	3.80	4.30
Exchangeable acidity (Meq/100 g soil)	3.36	0.56
EC (µs/cm)	103.80	89.30
CEC (cmol/kg soil)	94.30	82.24

## Data Availability

Data are contained within the article and Supplementary Materials.

## Supplementary Materials

The following supporting information can be downloaded at: https://www.mdpi.com/article/10.3390/plants14030311/s1, Table S1. Descriptions of 64 Ethiopian chickpea accessions used in the study; Table S2. Mean performance of 64 Ethiopian chickpea accessions for agro-morphological and yield traits grown at the Emdebir trial site under lime-treated and lime-untreated soil conditions; Table S3. Mean performance of 64 Ethiopian chickpea accessions for agro-morphological and yield traits grown at the Holetta trial site under lime-treated and lime-untreated soil conditions; Table S4. Stress tolerance indices of 64 Ethiopian chickpea accessions for seed yield assessed at the Emdebir and Holetta sites.
