# Supplementary material for: Genetic Variability of Ethiopian Chickpea (Cicer arietinum L.) Landraces for Acid Soil Tolerance
Source: Plants (Basel). 2025 Jan 21;14(3):311. doi: 10.3390/plants14030311 (PMC11819724; doi:10.3390/plants14030311)
Supplement: Supplementary file 1 [file plants-14-00311-s001.zip › Table S1.pdf]

Table S1. Descriptions of 64 Ethiopian chickpea accessions used in the study.

S/N	SAMPLE CODE	ACCESSION	REGION	ZONE	DISTRICT	LATITUDE	LONGITUDE	ALTITUDE	SEED SOURCE
1	ETC_41046	41046	AMHARA	NORTH GONDAR	CHILGA	12-33-00-N	37-04-00-E	2160	EBI
2	ETC_41086	41086	AMHARA	EAST GOJAM	GUZAMN	10-14-46-N	37-35-46-E	2181	EBI
3	ETC_41118	41118	OROMIYA	EAST SHEWA	ADA-A CHUKALA	08-45-00-N	38-59-00-E	1860	EBI
4	ETC_41128	41128	OROMIYA	EAST SHEWA	AKAKI	08-50-00-N	38-48-00-E	2130	EBI
5	ETC_41140	41140	OROMIYA	EAST SHEWA	LOME	08-34-00-N	39-19-00-E	2030	EBI
6	ETC_41175	41175	OROMIYA	WEST SHEWA	AMBO	08-59-00-N	37-51-00-E	1970	EBI
7	ETC_41184	41184	OROMIYA	WEST SHEWA	WALISONA GORO	08-32-00-N	37-59-00-E	2050	EBI
8	ETC_41186	41186	OROMIYA	WEST SHEWA	WALISONA GORO	08-27-00-N	37-55-00-E	1960	EBI
9	ETC_41191	41191	OROMIYA	WEST SHEWA	WALISONA GORO	08-35-00-N	37-59-00-E	2110	EBI
10	ETC_41200	41200	OROMIYA	WEST SHEWA	BECHO	08-45-00-N	38-19-00-E	2100	EBI
11	ETC_41215	41215	AMHARA	NORTH SHEWA	MORETNA JIRU	09-53-00-N	39-09-00-E	2640	EBI
12	ETC_41224	41224	AMHARA	EAST GOJAM	ENEMAY	10-30-00-N	38-09-00-E	2500	EBI
13	ETC_41237	41237	AMHARA	EAST GOJAM	ENEMAY	10-25-00-N	38-20-00-E	2400	EBI
14	ETC_41238	41238	AMHARA	EAST GOJAM	ENEMAY	10-25-00-N	38-20-00-E	2400	EBI
15	ETC_41248	41248	AMHARA	EAST GOJAM	ENEMAY	10-24-00-N	38-16-00-E	2350	EBI
16	ETC_41249	41249	AMHARA	EAST GOJAM	SHEBEL BERENTA	10-28-00-N	38-12-00-E	2350	EBI
17	ETC_41259	41259	AMHARA	EAST GOJAM	GONCHA SISO ENESE	10-53-00-N	38-13-00-E	2560	EBI
18	ETC_41265	41265	AMHARA	EAST GOJAM	HULET EJ ENESE	11-04-00-N	37-52-00-E	2450	EBI
19	ETC_41271	41271	AMHARA	WEST GOJAM	ADET	11-17-00-N	37-28-00-E	1880	EBI
20	ETC_41280	41280	AMHARA	NORTH GONDAR	GONDAR ZURIA	12-18-00-N	37-41-00-E	1940	EBI
21	ETC_41282	41282	AMHARA	BAHIR DAR SPECIAL	BAHIR DAR	11-35-00-N	37-21-00-E	1890	EBI
22	ETC_208985	208985	OROMIYA	NORTH SHEWA	BEREHNA ALELTU	09-08-00-N	38-54-00-E	2600	EBI
23	ETC_209008	209008	SNNP	HADIYA	BADAWACHO	07-10-06-N	37-54-57-E	1850	EBI
24	ETC_212477	212477	AMHARA	NORTH SHEWA	WEREMO WAJETUNA MID	10-14-00-N	39-00-00-E	1610	EBI
25	ETC_215667	215667	AMHARA	OROMIYA	BATI	11-11-00-N	39-50-00-E	2057	EBI
26	ETC_216853	216853	OROMIYA	ARSSI	TIYO	07-53-15-N	39-06-48-E	2487	EBI
27	ETC_231330	231330	OROMIYA	ARSSI	JEJU	08-23-10-N	39-41-35-E	2079	EBI

Seed Source: EBI- Ethiopian Biodiversity Institute; DZARC-Debrezeit Agricultural Research Center

Table S1. Cont…

<table>
<tr><th>S/N</th><th>SAMPLE CODE</th><th>ACCESSION</th><th>REGION</th><th>ZONE</th><th>DISTRICT</th><th>LATITUDE</th><th>LONGITUDE</th><th>ALTITUDE</th><th>SEED SOURCE</th></tr>
<tr><td>28</td><td>ETC_235031</td><td>235031</td><td>AMHARA</td><td>SOUTH WELLO</td><td>TEHULEDERE</td><td>11-13-00-N</td><td>39-38-00-E</td><td>1840</td><td>EBI</td></tr>
<tr><td>29</td><td>ETC_235035</td><td>235035</td><td>AMHARA</td><td>NORTH SHEWA</td><td>EFRATANA GIDIM</td><td>11-03 -00-N</td><td>39-43-00-E</td><td>1740</td><td>EBI</td></tr>
<tr><td>30</td><td>ETC_235393</td><td>235393</td><td>TIGRAY</td><td>CENTRAL TIGRAY</td><td>KOLA TEMBEN</td><td>13-42-00-N</td><td>39-05-00-E</td><td>1500</td><td>EBI</td></tr>
<tr><td>31</td><td>ETC_235394</td><td>235394</td><td>TIGRAY</td><td>CENTRAL TIGRAY</td><td>KOLA TEMBEN</td><td>13-36-00-N</td><td>39-09-00-E</td><td>1640</td><td>EBI</td></tr>
<tr><td>32</td><td>ETC_235396</td><td>235396</td><td>TIGRAY</td><td>CENTRAL TIGRAY</td><td>KOLA TEMBEN</td><td>13-39-51-N</td><td>38-54-58-E</td><td>1722</td><td>EBI</td></tr>
<tr><td>33</td><td>ETC_235398</td><td>235398</td><td>TIGRAY</td><td>SOUTHERN TIGRAY</td><td>MEKELE</td><td>13-29-37-N</td><td>39-27-57-E</td><td>2052</td><td>EBI</td></tr>
<tr><td>34</td><td>ETC_236462</td><td>236462</td><td>TIGRAY</td><td>CENTRAL TIGRAY</td><td>ENTICHO</td><td>14-16-44-N</td><td>39-09-04-E</td><td>2130</td><td>EBI</td></tr>
<tr><td>35</td><td>ETC_A_1_2016</td><td>A|DW|A|1|2016</td><td>AMHARA</td><td>SOUTH WELO</td><td>AMBASEL</td><td>11-29-33.866-N</td><td>039-36-52.358-E</td><td>1666</td><td>FARMERS</td></tr>
<tr><td>36</td><td>ETC_A_2_2016</td><td>A|DW|A|2|2016</td><td>AMHARA</td><td>SOUTH WELO</td><td>AMBASEL</td><td>11-29-43.908-N</td><td>039-36-45.162-E</td><td>1710</td><td>FARMERS</td></tr>
<tr><td>37</td><td>ETC_TD_4_2016</td><td>A|DW|TD|4|2016</td><td>AMHARA</td><td>AMHARA REGION OROMIA ZONE (KEMISSE)</td><td>TEHULE DERE</td><td>11-17-21.404-N</td><td>039-40-36.150-E</td><td>1994</td><td>FARMERS</td></tr>
<tr><td>38</td><td>ETC_K_3_2016</td><td>A|DW|K|3|2016</td><td>AMHARA</td><td>AMHARA REGION OROMIA ZONE (KEMISSE)</td><td>ARTUMA FURSE</td><td>10-34-34.377-N</td><td>039-55-10.620-E</td><td>1461</td><td>FARMERS</td></tr>
<tr><td>39</td><td>ETC_GN_1_2016</td><td>Ginchi 01/ Dendi</td><td>OROMIYA</td><td>WEST SHEWA</td><td>DENDI</td><td>09-00-59.19-N</td><td>038-12-40.633-E</td><td>2350</td><td>FARMERS</td></tr>
<tr><td>40</td><td>ETC_AM_1_2016</td><td>Ambo 01</td><td>OROMIYA</td><td>WEST SHEWA</td><td>AMBO</td><td>08-58-21.6-N</td><td>037-57-05.11-E</td><td>2394</td><td>FARMERS</td></tr>
<tr><td>41</td><td>ETC_BM_2_2016</td><td>Bambasi 02</td><td>BENISHANGUL-GUMUZ</td><td>ASOSA ZONE</td><td>BAMBASI</td><td>09-41-11.989-N</td><td>034-42-17.485-E</td><td>1462</td><td>FARMERS</td></tr>
<tr><td>42</td><td>ETC_WL_1_2016</td><td>Wollega LV</td><td>OROMIYA</td><td>KELEM WELLEGA</td><td>SEYO WOREDA</td><td>08-19-60- N</td><td>35-00-00- E</td><td>1850</td><td>FARMERS</td></tr>
<tr><td>43</td><td>ETC_HA_2_2016</td><td>A|DW|H|2|2016</td><td>AMHARA</td><td>SOUTH WELO</td><td>HABRU</td><td>11-34-50.012-N</td><td>039-39-46.052-E</td><td>1585</td><td>FARMERS</td></tr>
<tr><td>44</td><td>ETC_B_1_2016</td><td>O|B|1|2016</td><td>OROMIYA</td><td>SOUTH WEST SHEWA</td><td>BECHO</td><td>08-36-38.675-N</td><td>038-08-35.671-E</td><td>2216</td><td>FARMERS</td></tr>
<tr><td>45</td><td>ETC_B_2_2016</td><td>O|B|2|2016</td><td>OROMIYA</td><td>SOUTH WEST SHEWA</td><td>BECHO</td><td>08-38-46.372-N</td><td>038-08-26.360-E</td><td>2299</td><td>FARMERS</td></tr>
<tr><td>46</td><td>ETC_H_6_2016</td><td>O|H|6|2016</td><td>OROMIYA</td><td>WEST SHEWA</td><td>WELEMERA</td><td>09-00-49-N</td><td>038-27-52.9-E</td><td>2236</td><td>FARMERS</td></tr>
<tr><td>47</td><td>ETC_Il_1_2016</td><td>O|Illu|1|2016</td><td>OROMIYA</td><td>SOUTH WEST SHEWA</td><td>ILLU</td><td>08-47-32.424-N</td><td>038-19-28.011-E</td><td>2343</td><td>FARMERS</td></tr>
<tr><td>48</td><td>ETC_S_2_2016</td><td>O|S|2|2017</td><td>OROMIYA</td><td>SOUTH WEST SHEWA</td><td>SEBETA HAWAS</td><td>08-51-53.972-N</td><td>038-31-58.048-E</td><td>2060</td><td>FARMERS</td></tr>
<tr><td>49</td><td>ETC_S_3_2016</td><td>O|S|3|2018</td><td>OROMIYA</td><td>SOUTH WEST SHEWA</td><td>SEBETA HAWAS</td><td>08-50-55-N</td><td>038-30-14-E</td><td>2062</td><td>FARMERS</td></tr>
<tr><td>50</td><td>ETC_S_4_2016</td><td>O|S|4|2019</td><td>OROMIYA</td><td>SOUTH WEST SHEWA</td><td>SEBETA HAWAS</td><td>08-51-16.065-N</td><td>038-28-11.331-E</td><td>2078</td><td>FARMERS</td></tr>
<tr><td>51</td><td>ETC_SS_2_2016</td><td>O|S.S|2|2016</td><td>OROMIYA</td><td>SOUTH WEST SHEWA</td><td>SEDAN SODO</td><td>08-33-41.742-N</td><td>038-15-07.635-E</td><td>2287</td><td>FARMERS</td></tr>
<tr><td>52</td><td>ETC_K_6_2016</td><td>A|DW|K|6|2016</td><td>AMHARA</td><td>AMHARA REGION OROMIA ZONE (KEMISSE)</td><td>ARTUMA FURSE</td><td>10-30-54.255-N</td><td>039-56-51.626-E</td><td>1464</td><td>FARMERS</td></tr>
</table>

Seed Source: EBI- Ethiopian Biodiversity Institute; DZARC-Debrezeit Agricultural Research Center

Table S1. Cont…

S/N	SAMPLE CODE	ORIGIN	CLASSIFICATION	YEAR OF RELEASE	SEED SOURCE
53	Akaki	ICRISAT	Released variety	1995	DZARC
54	Dalota	ICRISAT	Released variety	2013	DZARC
55	Dubie	Ethiopia	Released variety	1978	DZARC
56	Dhera	ICARDA	Released variety	2016	DZARC
57	Ejere	ICARDA	Released variety	2005	DZARC
58	Kasech	ICRISAT	Released variety	2011	DZARC
59	Natoli	ICRISAT	Released variety	2007	DZARC
60	Yelbie	ICRISAT	Released variety	2006	DZARC
61	DZ-2012-CK-0032	-	Advanced line	-	DZARC
62	DZ-2012-CK-20113-2-0042	-	Advanced line	-	DZARC
63	DZ-2012-CK-0233	-	Advanced line	-	DZARC
64	DZ-2012-CK-0237	-	Advanced line	-	DZARC

Seed Source: DZARC-Debrezeit Agricultural Research Center
