# Supplementary material for: Genetic Variability of Ethiopian Chickpea (Cicer arietinum L.) Landraces for Acid Soil Tolerance
Source: Plants (Basel). 2025 Jan 21;14(3):311. doi: 10.3390/plants14030311 (PMC11819724; doi:10.3390/plants14030311)
Supplement: Supplementary file 1 [file plants-14-00311-s001.zip › Table S2.pdf]

Table S2. Mean performance of 64 Ethiopian chickpea accessions for agro-morphological and yield traits grown at the Emdebir trial site under lime-treated and lime-untreated soil conditions.

S/N	GEN	STC		DTF		DTM		PH		NPB		NPP		HSW		TSY	
		Limed	Unlimed	Limed	Unlimed	Limed	Unlimed	Limed	Unlimed	Limed	Unlimed	Limed	Unlimed	Limed	Unlimed	Limed	Unimed
1	DZ-2012-CK-0032	90	95	69	68	120	116	41	37	8	5	31	16	26	21	1519	849
2	DZ-2012-CK-20113-2-0042	43	45	66	68	115	118	38	32	6	4	37	21	19	16	784	407
3	ETC_41046	93	65	66	67	117	120	37	37	5	5	32	31	11	10	724	595
4	ETC_41086	90	90	67	63	118	118	38	31	7	3	44	16	10	9	837	231
5	DZ-2012-CK-0233	85	80	65	68	119	115	35	31	5	4	25	16	22	18	1202	654
6	DZ-2012-CK-0237	65	48	68	66	118	114	32	30	4	3	25	11	23	18	547	211
7	Kasech	100	85	68	66	122	121	46	40	5	4	9	6	20	19	497	296
8	ETC_41140	88	78	69	68	119	117	38	32	7	4	28	19	10	9	640	576
9	Dhera	88	85	71	70	125	125	55	50	5	4	9	15	23	25	370	399
10	Ejere	85	83	67	67	119	121	40	38	6	4	20	11	26	26	640	393
11	ETC_41118	93	70	65	65	117	115	40	38	4	4	37	22	9	10	1001	599
12	Dalota	83	90	66	68	118	118	39	37	7	4	24	18	24	25	1232	989
13	ETC_41128	78	75	67	67	119	116	39	35	7	4	38	29	10	9	1143	819
14	ETC_41175	65	90	67	66	118	117	41	36	9	4	47	28	9	8	818	1003
15	ETC_41184	90	78	68	64	114	118	41	37	7	5	41	30	11	10	1051	688
16	ETC_41186	78	70	67	68	118	118	42	39	9	5	34	21	10	9	906	572
17	ETC_41191	73	88	64	65	120	118	39	34	6	4	43	22	9	9	887	1496
18	ETC_41200	88	75	67	67	119	122	39	35	4	5	29	28	8	8	632	481
19	ETC_41215	98	80	65	66	118	118	40	34	6	6	45	34	10	9	1017	418
20	ETC_41224	68	70	69	69	124	119	37	37	7	6	33	25	9	9	702	480
21	ETC_41237	85	88	67	69	120	119	43	38	10	7	43	32	9	9	1382	957
22	ETC_41238	83	75	68	67	122	119	42	35	7	4	37	22	9	9	929	393
23	ETC_41249	80	88	68	67	119	119	41	36	9	6	40	27	10	10	898	782

STC = stand count; DTF = days to flowering; DTM = days to maturity; PH = plant height; NPB = number of primary branches per plant; NPP = number of pods per plant; HSW = hundred seed weight; and TSY = total seed yield, R^2 = coefficient of determination; CV = coefficient of variation

Table S2. Cont…

S/N	GEN	STC		DTF		DTM		PH		NPB		NPP		HSW		TSY	
		Limed	Unlimed	Limed	Unlimed	Limed	Unlimed	Limed	Unlimed	Limed	Unlimed	Limed	Unlimed	Limed	Unlimed	Limed	Unlimed
24	ETC_41259	88	65	68	67	120	117	42	36	7	5	45	23	10	10	1187	873
25	ETC_41265	80	68	67	67	117	118	39	36	7	5	47	23	11	9	1003	418
26	ETC_41280	90	88	67	67	118	116	43	36	6	4	47	22	10	10	1203	702
27	ETC_208985	88	90	67	68	118	118	38	33	7	6	38	32	9	8	993	604
28	ETC_212477	65	90	66	65	116	117	36	31	6	4	29	26	10	9	602	415
29	ETC_215667	85	70	66	66	117	119	42	33	7	4	34	27	9	9	1014	546
30	ETC_216853	65	58	66	64	118	116	35	28	6	3	42	25	10	9	821	325
31	ETC_235031	88	85	68	68	116	115	42	31	7	5	38	22	10	10	1329	691
32	ETC_235035	80	80	52	65	117	115	37	36	5	4	38	25	10	10	889	466
33	ETC_231330	90	80	67	64	117	115	38	35	6	5	38	38	13	13	1255	1084
34	ETC_235393	78	85	64	65	117	115	37	35	6	5	44	29	10	9	880	663
35	ETC_235394	60	60	67	64	117	116	40	35	7	3	47	29	10	9	1042	713
36	ETC_235396	78	73	67	65	114	115	37	35	6	4	44	33	11	9	897	621
37	ETC_235398	78	83	65	66	116	113	36	35	5	3	25	22	10	10	806	583
38	ETC_236462	68	83	64	68	116	116	39	35	5	6	32	38	10	9	937	1138
39	ETC_41282	93	55	68	68	119	118	41	35	7	5	45	24	9	9	1004	882
40	ETC_A_1_2016	90	78	66	66	117	115	35	33	8	5	59	21	10	9	1311	680
41	ETC_A_2_2016	88	100	65	67	117	116	37	33	6	4	39	15	10	10	1217	947
42	ETC_TD_4_2016	73	55	67	67	115	113	36	29	5	4	36	20	10	9	964	948
43	ETC_K_3_2016	80	55	66	67	117	114	35	32	6	4	28	39	11	10	873	1518
44	ETC_GN_1_2016	90	68	66	68	117	116	38	32	6	4	33	21	10	9	1057	789
45	ETC_AM_1_2016	93	88	64	67	118	118	40	36	6	4	32	24	10	9	1095	625
46	ETC_BM_2_2016	90	88	67	67	113	114	37	32	7	5	32	28	10	10	880	916
47	ETC_209008	85	78	66	69	120	117	41	35	8	3	46	30	9	10	1040	693
48	Dubie	100	90	68	67	119	117	38	36	7	3	26	22	16	16	896	687
49	ETC_WL_1_2016	68	60	68	67	117	115	42	34	8	5	58	38	10	10	1332	1410

STC = stand count; DTF = days to flowering; DTM = days to maturity; PH = plant height; NPB = number of primary branches per plant; NPP = number of pods per plant; HSW = hundred seed weight; and TSY = total seed yield, R^2 = coefficient of determination; CV = coefficient of variation

Table S2. Cont…

S/N	GEN	STC		DTF		DTM		PH		NPB		NPP		HSW		TSY	
		Limed	Unlimed	Limed	Unlimed	Limed	Unlimed	Limed	Unlimed	Limed	Unlimed	Limed	Unlimed	Limed	Unlimed	Limed	Unlimed
50	ETC_HA_2_2016	80	80	67	67	115	114	40	40	6	4	41	47	10	9	743	1567
51	Natoli	75	75	71	70	122	120	39	40	6	5	26	33	25	22	1098	1225
52	ETC_B_1_2016	95	100	63	68	117	118	41	40	6	7	47	60	11	10	1171	1657
53	ETC_B_2_2016	73	90	64	68	118	118	38	38	7	5	42	35	10	8	746	850
54	ETC_41248	75	80	68	68	122	120	39	39	7	6	39	25	10	9	804	497
55	ETC_H_6_2016	85	63	66	67	115	120	43	32	6	2	41	17	12	9	1127	298
56	ETC_41271	90	55	67	65	119	115	42	35	8	4	37	24	10	9	944	738
57	ETC_Il_1_2016	80	85	65	67	117	117	39	39	5	6	30	28	11	8	682	899
58	ETC_S_2_2016	78	98	70	73	125	123	35	36	8	4	24	20	20	19	605	753
59	ETC_S_3_2016	93	100	70	71	122	124	38	38	4	3	12	12	19	20	290	501
60	ETC_S_4_2016	93	88	67	68	118	117	38	35	5	4	33	26	11	10	887	652
61	ETC_SS_2_2016	83	93	66	67	116	120	41	34	6	3	26	14	7	9	641	575
62	ETC_K_6_2016	93	75	66	68	118	116	34	36	7	5	30	43	10	9	770	819
63	Yelebe	90	85	66	68	120	117	43	41	7	4	27	16	22	24	937	722
64	Akaki	98	85	69	71	120	124	38	33	7	3	27	18	16	16	944	769
	Mean	82.83	78.38	66.58	67.09	118.2	117.47	39.25	35.34	6.44	4.39	35.23	25.22	12.48	11.72	926.2	730.42
	LSD(0.05)	20.61	24.86	6.34	2.87	3.94	4.08	6.14	562	2.02	1.28	8.97	5.14	3.84	3.47	269.4	165.56
	CV (%)	12.42	15.82	4.76	2.14	1.66	1.73	7.79	7.92	15.6	14.61	12.67	10.18	15.25	14.72	14.48	11.28
	PR (%)	5.38		-0.77		0.62		9.95		31.8		28.43		6.13		21.13	
	SE.M	1.15	1.56	0.29	0.19	0.26	0.26	0.36	0.39	0.14	0.11	0.95	0.84	0.47	0.45	23.46	29.52

STC = stand count; DTF = days to flowering; DTM = days to maturity; PH = plant height; NPB = number of primary branches per plant; NPP = number of pods per plant; HSW = hundred seed weight; and TSY = total seed yield, R^2 = coefficient of determination; CV = coefficient of variation
