# Supplementary material for: Genetic Variability of Ethiopian Chickpea (Cicer arietinum L.) Landraces for Acid Soil Tolerance
Source: Plants (Basel). 2025 Jan 21;14(3):311. doi: 10.3390/plants14030311 (PMC11819724; doi:10.3390/plants14030311)
Supplement: Supplementary file 1 [file plants-14-00311-s001.zip › Table S3.pdf]

Table S3. Mean performance of 64 Ethiopian chickpea accessions for agro-morphological and yield traits grown at the Holetta trial site under lime-treated and lime-untreated soil conditions.

S/N	GEN	STC		DTF		DTM		PH		NPB		NPP		HSW		TSY	
		Limed	Unlimed	Limed	Unlimed	Limed	Unlimed	Limed	Unlimed	Limed	Unlimed	Limed	Unlimed	Limed	Unlimed	Limed	Unlimed
1	DZ-2012-CK-0032	50	45	54	57	126	126	36	28	6	6	10	16	10	24	288	260
2	DZ-2012-CK-20113-2-0042	25	15	59	63	122	128	27	26	4	4	4	7	23	19	104	99
3	ETC_41046	50	13	53	56	124	125	36	22	6	5	12	12	5	26	150	136
4	ETC_41086	40	25	62	63	130	129	41	42	5	5	6	18	20	16	60	191
5	DZ-2012-CK-0233	25	43	59	60	125	129	35	29	6	7	12	22	16	22	301	463
6	DZ-2012-CK-0237	8	10	60	71	125	128	27	16	5	3	2	7	5	26	17	86
7	Kasech	48	20	64	69	130	128	31	22	4	3	2	6	4	26	13	197
8	ETC_41140	55	48	55	57	125	124	36	33	4	6	25	25	11	10	641	369
9	Dhera	45	38	62	64	135	134	39	35	4	5	3	6	2	30	18	65
10	Ejere	25	48	53	57	120	124	31	25	4	4	5	8	22	16	110	85
11	ETC_41118	30	43	57	56	122	123	36	31	5	5	24	29	10	11	438	384
12	Dalota	23	43	54	56	120	126	39	32	7	5	26	23	17	26	525	463
13	ETC_41128	43	58	57	58	126	129	37	33	4	7	22	28	10	10	392	297
14	ETC_41175	20	30	62	56	125	127	33	27	6	6	15	20	10	9	148	304
15	ETC_41184	18	25	58	56	125	124	34	26	6	4	19	13	11	9	241	131
16	ETC_41186	38	28	60	54	131	124	32	27	5	7	8	14	9	10	182	200
17	ETC_41191	20	53	55	59	123	125	33	28	5	4	16	15	9	10	188	269
18	ETC_41200	20	40	57	57	120	126	31	30	5	6	8	28	10	9	60	256
19	ETC_41215	33	35	57	59	124	125	32	27	6	6	21	22	12	10	356	224
20	ETC_41224	50	68	59	58	123	121	38	35	6	7	25	26	11	11	794	467
21	ETC_41237	45	60	60	59	127	124	37	32	7	6	29	26	11	11	1052	534
22	ETC_41238	48	38	61	61	124	128	38	30	7	6	39	23	11	10	648	300

STC = stand count; DTF = days to flowering; DTM = days to maturity; PH = plant height; NPB = number of primary branches per plant; NPP = number of pods per plant; HSW = hundred seed weight; and TSY = total seed yield, R^2 = coefficient of determination; CV = coefficient of variation

Table S3. Cont…

S/N	GEN	STC		DTF		DTM		PH		NPB		NPP		HSW		TSY	
		Limed	Unlimed	Limed	Unlimed	Limed	Unlimed	Limed	Unlimed	Limed	Unlimed	Limed	Unlimed	Limed	Unlimed	Limed	Unlimed
23	ETC_41249	38	38	64	61	127	125	36	29	7	5	21	18	12	11	412	251
24	ETC_41259	30	23	61	60	122	124	35	27	9	5	22	21	11	10	510	306
25	ETC_41265	28	58	57	58	124	130	32	30	6	6	14	25	11	11	124	503
26	ETC_41280	33	63	55	56	121	125	35	33	5	7	17	35	12	11	269	511
27	ETC_208985	33	30	57	58	121	126	31	25	4	5	14	22	11	11	298	333
28	ETC_212477	28	58	58	56	125	124	33	28	7	5	27	20	12	12	487	483
29	ETC_215667	58	43	60	61	124	127	34	37	5	9	22	37	10	11	460	461
30	ETC_216853	48	30	54	58	125	123	34	27	7	6	43	31	11	11	891	346
31	ETC_235031	43	35	60	60	132	124	36	31	8	4	38	21	11	10	750	251
32	ETC_235035	28	73	56	57	121	123	28	23	6	4	33	23	10	11	483	438
33	ETC_231330	48	53	53	57	124	123	28	30	7	4	21	25	13	12	492	474
34	ETC_235393	48	23	53	58	125	124	32	30	6	6	21	27	11	10	331	251
35	ETC_235394	43	25	52	58	123	126	33	33	5	8	24	35	12	11	615	305
36	ETC_235396	45	23	55	56	121	126	37	26	5	3	34	18	11	10	980	147
37	ETC_235398	58	58	52	55	121	123	33	28	7	4	32	25	11	11	753	457
38	ETC_236462	40	38	54	56	125	124	32	27	6	5	27	18	11	11	480	309
39	ETC_41282	65	23	58	59	123	120	37	30	8	7	32	21	10	10	555	230
40	ETC_A_1_2016	45	33	55	56	123	128	34	32	7	6	34	30	11	11	656	452
41	ETC_A_2_2016	35	20	58	56	124	127	26	12	4	2	16	5	8	10	298	112
42	ETC_TD_4_2016	38	33	55	56	122	125	31	31	6	7	28	35	10	11	430	341
43	ETC_K_3_2016	55	50	54	57	127	125	34	31	7	9	40	36	10	10	1189	450
44	ETC_GN_1_2016	43	65	59	55	126	127	33	32	5	6	19	26	11	11	441	392
45	ETC_AM_1_2016	38	63	55	54	124	121	36	33	6	7	35	18	11	11	885	381
46	ETC_BM_2_2016	28	58	58	58	122	123	34	29	5	5	15	25	12	8	472	485
47	ETC_209008	20	45	58	58	126	130	27	29	4	6	9	14	20	13	150	189

STC = stand count; DTF = days to flowering; DTM = days to maturity; PH = plant height; NPB = number of primary branches per plant; NPP = number of pods per plant; HSW = hundred seed weight; and TSY = total seed yield, R^2 = coefficient of determination; CV = coefficient of variation

Table S3. Cont…

S/N	GEN	STC		DTF		DTM		PH		NPB		NPP		HSW		TSY	
		Limed	Unlimed	Limed	Unlimed	Limed	Unlimed	Limed	Unlimed	Limed	Unlimed	Limed	Unlimed	Limed	Unlimed	Limed	Unlimed
48	Dubie	28	43	63	57	128	126	28	30	4	6	23	29	14	17	451	479
49	ETC_WL_1_2016	33	43	55	56	125	127	28	27	7	7	20	20	11	10	575	251
50	ETC_HA_2_2016	65	45	56	59	124	122	35	28	7	5	16	16	10	10	610	274
51	Natoli	43	48	62	62	127	133	34	29	4	4	14	12	25	21	553	317
52	ETC_B_1_2016	53	43	56	57	125	127	41	37	5	6	34	23	12	11	859	502
53	ETC_B_2_2016	43	53	56	57	128	126	37	32	5	6	34	29	11	10	831	389
54	ETC_41248	50	35	60	61	126	130	37	29	7	7	39	24	11	11	1002	317
55	ETC_H_6_2016	40	38	57	57	128	129	34	30	6	6	44	26	11	11	797	410
56	ETC_41271	30	65	58	58	122	123	31	27	5	5	25	14	10	11	382	306
57	ETC_Il_1_2016	40	43	56	59	124	126	68	28	5	6	22	19	12	11	698	360
58	ETC_S_2_2016	55	58	61	62	128	126	30	24	5	5	15	11	19	21	411	156
59	ETC_S_3_2016	43	60	62	66	136	122	34	32	4	4	15	7	20	28	446	196
60	ETC_S_4_2016	48	53	57	60	121	121	30	30	5	6	21	25	10	10	500	455
61	ETC_SS_2_2016	35	63	57	57	123	123	30	31	4	5	30	24	10	10	706	325
62	ETC_K_6_2016	43	43	55	57	121	128	31	25	6	6	35	24	11	10	788	267
63	Yelebe	35	25	54	59	128	130	33	25	6	6	13	12	4	25	126	77
64	Akaki	23	40	67	63	130	125	31	26	3	3	19	9	14	7	728	64
	Mean	38.78	41.95	57.52	58.55	124.91	125.69	33.94	28.89	5.58	5.48	21.72	20.77	11.67	13.34	478.13	309.11
	LSD(0.05)	13.01	13.92	5.26	3.17	6.12	5.12	7.14	7.32	2.19	1.88	6.47	6.27	4.43	3.91	130.56	125.37
	CV(%)	16.81	16.64	4.56	2.7	2.44	2.03	10.49	12.63	19.69	17.4	14.85	15.08	18.97	14.57	27.18	20.2
	PR (%)	-8.18		-1.79		-0.63		14.87		1.68		4.39		-14.32		35.35	
	SE.M	1.14	1.39	0.34	0.3	0.36	0.34	0.55	0.47	0.13	0.14	0.97	0.75	0.39	0.52	13.29	12.37

STC = stand count; DTF = days to flowering; DTM = days to maturity; PH = plant height; NPB = number of primary branches per plant; NPP = number of pods per plant; HSW = hundred seed weight; and TSY = total seed yield, R^2 = coefficient of determination; CV = coefficient of variation
