# Supplementary material for: Genetic Variability of Ethiopian Chickpea (Cicer arietinum L.) Landraces for Acid Soil Tolerance
Source: Plants (Basel). 2025 Jan 21;14(3):311. doi: 10.3390/plants14030311 (PMC11819724; doi:10.3390/plants14030311)
Supplement: Supplementary file 1 [file plants-14-00311-s001.zip › Table S4.pdf]

Table S4. Stress tolerance indices of 64 Ethiopian chickpea accessions for seed yield assessed at the Emdebir and Holetta sites.

		EMDEBIR					HOLETTA				
S/N	GEN	TSYLTP	TSYLUTP	TOL	YSI	STI	TSYLTP	TSYLUTP	TOL	YSI	STI
1	DZ-2012-CK-0032	1519	849	670	0.56	1.5	288	260	28	0.9	0.33
2	DZ-2012-CK-20113-2-0042	784	407	377	0.52	0.37	104	99	5	0.95	0.05
3	ETC_41046	724	595	129	0.82	0.5	150	136	14	0.91	0.09
4	ETC_41086	837	231	606	0.28	0.23	60	191	-131	3.18	0.05
5	DZ-2012-CK-0233	1202	654	548	0.54	0.92	301	463	-162	1.54	0.61
6	DZ-2012-CK-0237	547	211	336	0.39	0.13	17	86	-69	5.06	0.01
7	Kasech	497	296	201	0.6	0.17	13	197	-184	15.15	0.01
8	ETC_41140	640	576	64	0.9	0.43	641	369	272	0.58	1.03
9	Dhera	370	399	-29	1.08	0.17	18	65	-47	3.61	0.01
10	Ejere	640	393	247	0.61	0.29	110	85	25	0.77	0.04
11	ETC_41118	1001	599	402	0.6	0.7	438	384	54	0.88	0.74
12	Dalota	1232	989	243	0.8	1.42	525	463	62	0.88	1.06
13	ETC_41128	1143	819	324	0.72	1.09	392	297	95	0.76	0.51
14	ETC_41175	818	1003	-185	1.23	0.96	148	304	-156	2.05	0.2
15	ETC_41184	1051	688	363	0.65	0.84	241	131	110	0.54	0.14
16	ETC_41186	906	572	334	0.63	0.6	182	200	-18	1.1	0.16
17	ETC_41191	887	1496	-609	1.69	1.55	188	269	-81	1.43	0.22
18	ETC_41200	632	481	151	0.76	0.35	60	256	-196	4.27	0.07
19	ETC_41215	1017	418	599	0.41	0.5	356	224	132	0.63	0.35
20	ETC_41224	702	480	222	0.68	0.39	794	467	327	0.59	1.62
21	ETC_41237	1382	957	425	0.69	1.54	1052	534	518	0.51	2.46
22	ETC_41238	929	393	536	0.42	0.43	648	300	348	0.46	0.85
23	ETC_41249	898	782	116	0.87	0.82	412	251	161	0.61	0.45
24	ETC_41259	1187	873	314	0.74	1.21	510	306	204	0.6	0.68
25	ETC_41265	1003	418	585	0.42	0.49	124	503	-379	4.06	0.27
26	ETC_41280	1203	702	501	0.58	0.98	269	511	-242	1.9	0.6
27	ETC_208985	993	604	389	0.61	0.7	298	333	-35	1.12	0.43
28	ETC_212477	602	415	187	0.69	0.29	487	483	4	0.99	1.03
29	ETC_215667	1014	546	468	0.54	0.65	460	461	-1	1	0.93
30	ETC_216853	821	325	496	0.4	0.31	891	346	545	0.39	1.35
31	ETC_235031	1329	691	638	0.52	1.07	750	251	499	0.33	0.82
32	ETC_235035	889	466	423	0.52	0.48	483	438	45	0.91	0.93

TSYLTP: Total seed yield from lime treated plot; TSYLUTP: Total seed yield from lime untreated plot; TOL: Tolerance level; YSI: Yield stability index; and STI: Stress tolerance index.

Table S4. Cont…

		EMDEBIR					HOLETTA				
S/N	GEN	TSYLTP	TSYLUTP	TOL	YSI	STI	TSYLTP	TSYLUTP	TOL	YSI	STI
33	ETC_231330	1255	1084	171	0.86	1.59	492	474	18	0.96	1.02
34	ETC_235393	880	663	217	0.75	0.68	331	251	80	0.76	0.36
35	ETC_235394	1042	713	329	0.68	0.87	615	305	310	0.5	0.82
36	ETC_235396	897	621	276	0.69	0.65	980	147	833	0.15	0.63
37	ETC_235398	806	583	223	0.72	0.55	753	457	296	0.61	1.51
38	ETC_236462	937	1138	-201	1.21	1.24	480	309	171	0.64	0.65
39	ETC_41282	1004	882	122	0.88	1.03	555	230	325	0.41	0.56
40	ETC_A_1_2016	1311	680	631	0.52	1.04	656	452	204	0.69	1.3
41	ETC_A_2_2016	1217	947	270	0.78	1.34	298	112	186	0.38	0.15
42	ETC_TD_4_2016	964	948	16	0.98	1.07	430	341	89	0.79	0.64
43	ETC_K_3_2016	873	1518	-645	1.74	1.54	1189	450	739	0.38	2.34
44	ETC_GN_1_2016	1057	789	268	0.75	0.97	441	392	49	0.89	0.76
45	ETC_AM_1_2016	1095	625	470	0.57	0.8	885	381	504	0.43	1.47
46	ETC_BM_2_2016	880	916	-36	1.04	0.94	472	485	-13	1.03	1
47	ETC_209008	1040	693	347	0.67	0.84	150	189	-39	1.26	0.12
48	Dubie	896	687	209	0.77	0.72	451	479	-28	1.06	0.94
49	ETC_WL_1_2016	1332	1410	-78	1.06	2.19	575	251	324	0.44	0.63
50	ETC_HA_2_2016	743	1567	-824	2.11	1.36	610	274	336	0.45	0.73
51	Natoli	1098	1225	-127	1.12	1.57	553	317	236	0.57	0.77
52	ETC_B_1_2016	1171	1657	-486	1.42	2.26	859	502	357	0.58	1.89
53	ETC_B_2_2016	746	850	-104	1.14	0.74	831	389	442	0.47	1.41
54	ETC_41248	804	497	307	0.62	0.47	1002	317	685	0.32	1.39
55	ETC_H_6_2016	1127	298	829	0.26	0.39	797	410	387	0.51	1.43
56	ETC_41271	944	738	206	0.78	0.81	382	306	76	0.8	0.51
57	ETC_Il_1_2016	682	899	-217	1.32	0.71	698	360	338	0.52	1.1
58	ETC_S_2_2016	605	753	-148	1.24	0.53	411	156	255	0.38	0.28
59	ETC_S_3_2016	290	501	-211	1.73	0.17	446	196	250	0.44	0.38
60	ETC_S_4_2016	887	652	235	0.74	0.67	500	455	45	0.91	1
61	ETC_SS_2_2016	641	575	66	0.9	0.43	706	325	381	0.46	1
62	ETC_K_6_2016	770	819	-49	1.06	0.74	788	267	521	0.34	0.92
63	Yelebe	937	722	215	0.77	0.79	126	77	49	0.61	0.04
64	Akaki	944	769	175	0.81	0.85	728	64	664	0.09	0.2
	Mean	926	730	196	1	1	478	309	169	1	1

TSYLTP: Total seed yield from lime treated plot; TSYLUTP: Total seed yield from lime untreated plot; TOL: Tolerance level; YSI: Yield stability index; and STI: Stress tolerance index.
